# The Emerging Role of Central and Peripheral Immune Systems in Neurodegenerative Diseases

**DOI:** 10.3389/fnagi.2022.872134

**Published:** 2022-04-25

**Authors:** Xin Zang, Si Chen, JunYao Zhu, Junwen Ma, Yongzhen Zhai

**Affiliations:** ^1^Department of Infectious Disease, Shengjing Hospital of China Medical University, Shenyang, China; ^2^Department of Neurology, the Fourth Affiliated Hospital of China Medical University, Shenyang, China

**Keywords:** peripheral immune system, central nervous system, neurodegenerative diseases, Amyotrophic lateral sclerosis, Alzheimer's disease, Parkinson's disease

## Abstract

For decades, it has been widely believed that the blood–brain barrier (BBB) provides an immune privileged environment in the central nervous system (CNS) by blocking peripheral immune cells and humoral immune factors. This view has been revised in recent years, with increasing evidence revealing that the peripheral immune system plays a critical role in regulating CNS homeostasis and disease. Neurodegenerative diseases are characterized by progressive dysfunction and the loss of neurons in the CNS. An increasing number of studies have focused on the role of the connection between the peripheral immune system and the CNS in neurodegenerative diseases. On the one hand, peripherally released cytokines can cross the BBB, cause direct neurotoxicity and contribute to the activation of microglia and astrocytes. On the other hand, peripheral immune cells can also infiltrate the brain and participate in the progression of neuroinflammatory and neurodegenerative diseases. Neurodegenerative diseases have a high morbidity and disability rate, yet there are no effective therapies to stop or reverse their progression. In recent years, neuroinflammation has received much attention as a therapeutic target for many neurodegenerative diseases. In this review, we highlight the emerging role of the peripheral and central immune systems in neurodegenerative diseases, as well as their interactions. A better understanding of the emerging role of the immune systems may improve therapeutic strategies for neurodegenerative diseases.

## Introduction

Neurodegenerative diseases are characterized by progressive dysfunction and the loss of neurons in the CNS. In recent years, the incidence of neurodegenerative diseases associated with aging, especially Alzheimer's disease (AD), has increased exponentially with the aging of the global population (Schwartz and Deczkowska, 2016). However, there are no effective therapies to stop or reverse the progression of neurodegenerative diseases. Studies have shown that the aggregation and deposition of misfolded proteins play key roles in neurodegenerative diseases (Hartl, 2017; Abdel-Nour et al., 2019). In the last decade, research on the role of the immune system in neurodegenerative diseases has progressed. Both the innate and adaptive immune systems have been shown to be involved in the inflammatory mechanisms associated with the accumulation of misfolded proteins in the brain (Ciccocioppo et al., 2020).

The central immune system is composed of neurons, glial cells as well as other immune cells. Traditionally, studies have considered the peripheral and central immune systems to be separate processes because the BBB blocks peripheral immune cells and humoral immune factors (Jeon et al., 2021). However, there is increasing evidence that peripheral immune system plays an important role in neuropathology. Peripheral immune cells can participate in the progression of neuroinflammatory and neurodegenerative diseases by infiltrating the brain (Greenhalgh et al., 2020). In addition, peripherally released cytokines can cross the BBB to cause direct neurotoxicity and contribute to the activation of glial cells (Fani Maleki and Rivest, 2019). Activated glial cells lead to further secretion of pro-inflammatory chemokines and cytokines, thereby recruiting more immune cells to the CNS (Prinz and Priller, 2017; Vainchtein and Molofsky, 2020). Although neurodegenerative diseases have different etiologies and pathogeneses, they all share the characteristic of neuroinflammation. In recent years, neuroinflammation has received significant interest as a potential therapeutic target for many neurodegenerative diseases. In this review, we summarize the emerging role of the peripheral and central immune systems in neurodegenerative diseases, as well as their interactions, which may have important implications for understanding the pathogenesis and progression and provide new ideas for therapeutic strategies to treat neurodegenerative diseases.

## The Central Nervous System

### Microglia

Microglia are long-lived resident macrophages in the CNS. As part of CNS homeostasis, microglia remain quiescent under physiological conditions and perform extremely strong immune surveillance through highly mobile processes (Nimmerjahn et al., 2005). This continuous state of movement enables microglia to respond rapidly to neuropathological changes. On the one hand, these cells play a phagocytic role, engulfing pathogens and cell debris that invade the brain. On the other hand, microglia are also able to transform into an activated phenotype under certain stimuli, accompanied by transcriptional changes to perform inflammatory functions (Amor et al., 2022).

**Figure 1:**
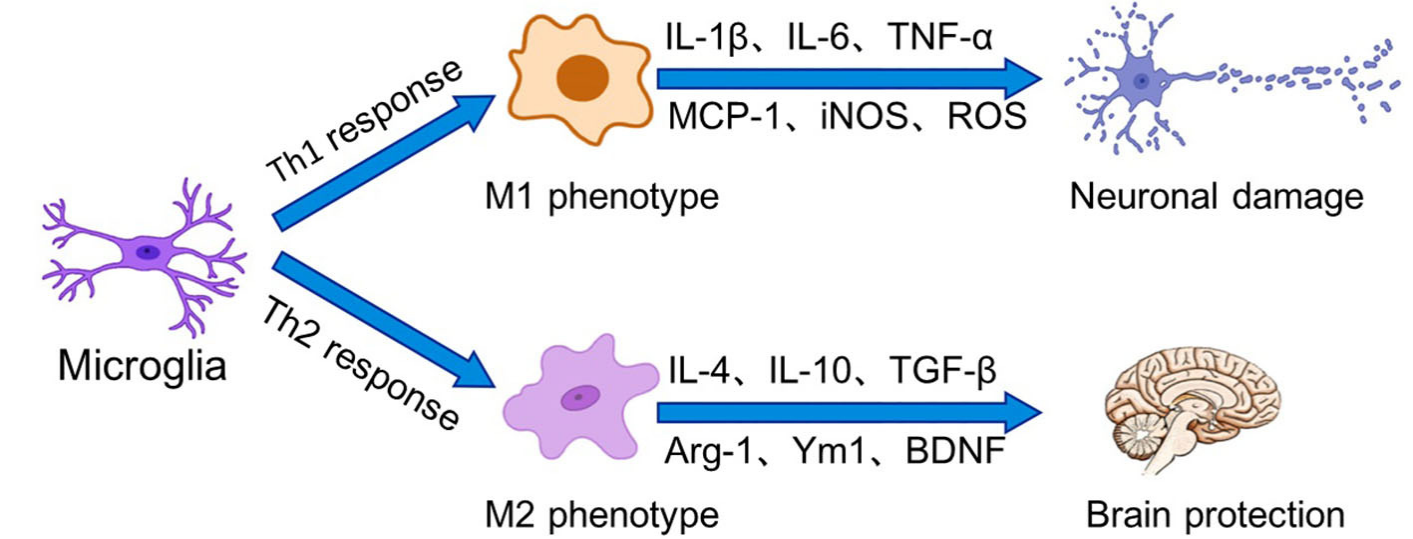
Microglia perform surveillance functions of the extracellular environment of the brain in the resting state. In the presence of stressors, microglia can be activated into two states, which have been traditionally divided into M1 (classical activation) and M2 (alternative activation). Binding of receptors on microglia to Th1 cytokines drives microglia polarization to the M1 phenotype, leading to increased inflammation and oxidative stress as well as BBB dysfunction. In contrast, Th2 cytokines prompt microglia to polarize to the M2 phenotype. M2 phenotype microglia promote tissue repair and regeneration through the production of anti-inflammatory cytokines and neurotrophic factors for to exert neuroprotective effects.

In general, the activation of microglia can be simplified into two states, which have been traditionally divided into M1 (classic activation) and M2 (alternative activation) (Figure 1). Sustained activation of the M1 phenotype results in the secretion of excessive amounts of pro-inflammatory cytokines and neurotoxic molecules, which in turn damage the organism. In contrast, M2 microglia promote tissue repair and regeneration through the production of anti-inflammatory cytokines and neurotrophic factors to exert neuroprotective effects. In recent years, however, it has been shown that activated microglia express canonical gene products associated with the M1 and M2 phenotypes (Rahimian et al., 2021). In neuropathological conditions, microglial activation falls on a continuum, and these cells exhibit a mixed phenotype mediated by a complex cascade of surrounding signals. Classifying microglia based on M1 and M2 polarization states is not sufficient to describe the multiple states of microglial activation (Ransohoff, 2016).

Recent studies have shown that microglia can make direct connections with different regions of neurons, which is a more precise modulation that regulates neuronal responses and cell fate (Cserép et al., 2020, 2021). Microglia can sense ATP produced by neuronal activation and break ATP down into adenosine, which inhibits adenosine receptors on the surface of active neurons, thereby inhibiting excessive neuronal activation and inducing negative feedback control of neuronal activity (Badimon et al., 2020). In some neurodegenerative conditions, however, microglia lose this ability to sense ATP molecules and produce adenosine. Therefore, in-depth research on the mechanism of microglia-neuronal communication may provide new ideas for the treatment of certain neurodegenerative diseases.

In fact, it is widely believed that microglia are able to not only interact with the immune components of the CNS but also crosstalk with peripheral immune components that infiltrate the CNS (Liu et al., 2020). Moreover, microglia have many important but not yet fully understood roles in protecting the brain from disease, and this offers the possibility of developing targeted molecular therapies. However, under pathological conditions, activated microglia can exert deleterious effects, and microglia can mediate the onset and development of neuro-inflammatory responses through a range of transcription factors and multiple cellular signaling pathways (Cai et al., 2018). Therefore, targeting the microglial inflammatory signaling pathway may be a potential approach to treat neurodegenerative diseases.

### Astrocytes

**Figure 2:**
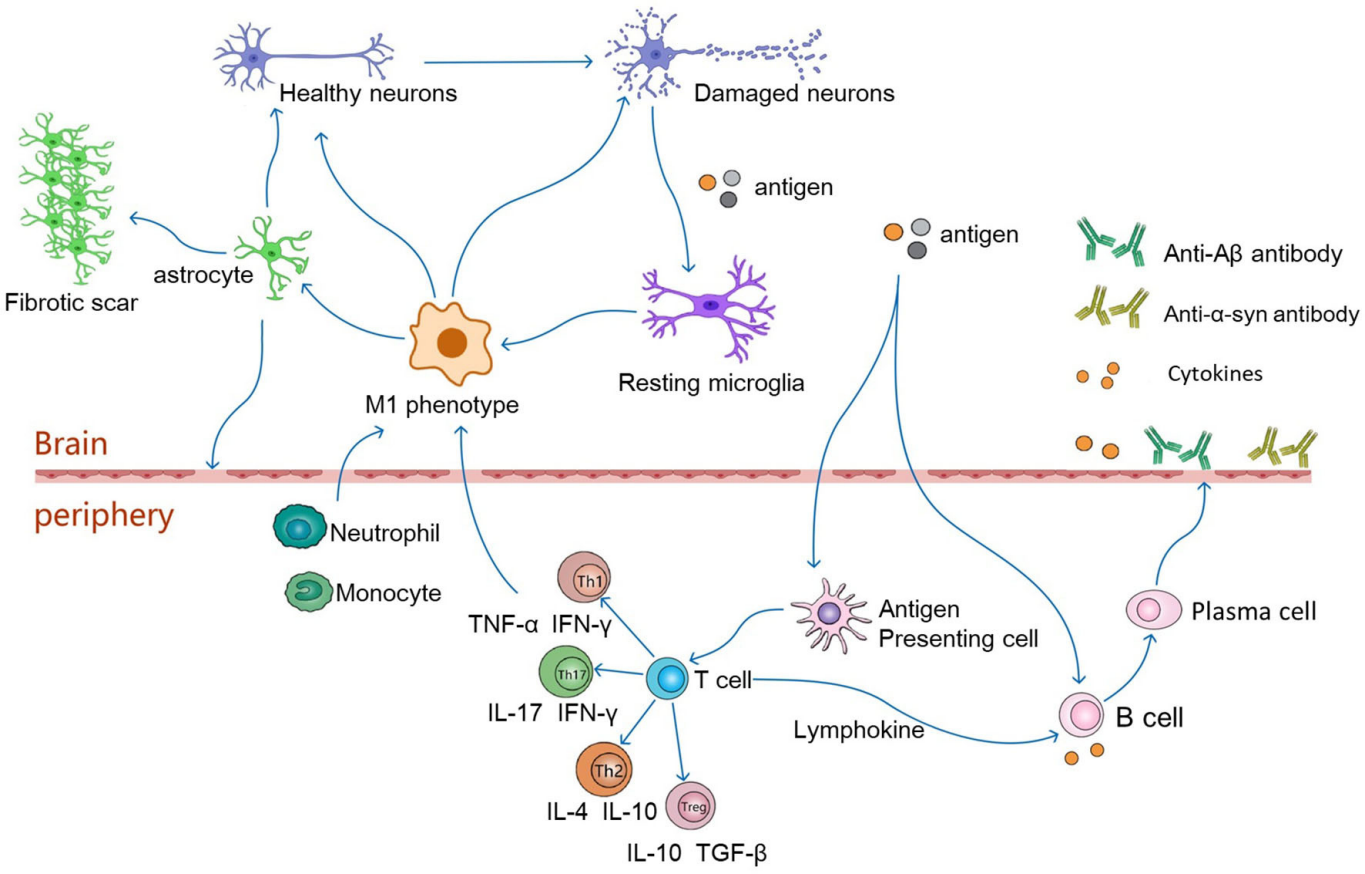
In pathological conditions, damaged neurons release autoantigens to activate resting microglia, which differentiate into the pro-inflammatory phenotype of M1. The M1 phenotype microglia secrete pro-inflammatory factors(IL-6, TNF-α, IFN-γ) to activate astrocytes, which induce the activation of A1 phenotype astrocytes. Reactive astrocytes contribute to the formation of glial scarring. It also leads to destruction of the BBB, which in turn leads to infiltration of CNS by peripheral immune cells. Antigens may enter peripheral lymphoid tissue, where they are presented by antigen-presenting cells to naive T cells, which differentiate into antigen-specific T effector cells (Th1, Th2, Th17 or Tregs). These cells then secrete anti-inflammatory or pro-inflammatory factors to regulate neuronal survival. Th1 and Th17 cells cross the BBB, produce neurotoxic and pro-inflammatory factors that interact with glial cells, leading directly to neuroinflammation and damage to motor neurons. Th2 and Tregs migrate from the periphery to the central nervous system and interact with glial cells to function as motor neuron protectors. In addition, antigens can directly stimulate B cells, which are activated to produce pro-inflammatory factors that travel along blood vessels to the brain and participate in neurodegeneration. Activated T cells secrete lymphokines to activate B cells, which proliferate and differentiate into plasma cells. Plasma cells can further produce cytokines and antibodies, such as anti-Aβ or anti-α-synuclein antibodies, which cross the blood-brain barrier into the brain to alleviate neuronal degeneration.

Astrocytes provide nutritional support for neurons, regulate the metabolic balance of the nervous system, and play an important role in promoting the formation and function of synapses and maintaining the structure of the brain and the BBB (Alvarez et al., 2011; Jeon et al., 2021). In addition, an increasing number of studies have proven that astrocytes are a double-edged sword. Similar to the M1 and M2 polarization states of macrophages, murine reactive astrocytes are defined as A1 and A2 (Liddelow et al., 2017). The A1 astrocytes are pro-inflammatory and neurotoxic and are associated with the progression of neurodegenerative diseases. In contrast, A2 astrocytes have a neuroprotective function. In a mouse cell model, microglia express interleukin (IL)-1α, tumor necrosis factor (TNF), and complement component 1q (C1q) in response to lipopolysaccharide (LPS) stimulation, and the combined effects of these cytokines are critical for the activation of A1-type astrocytes (Liddelow et al., 2017; Ridler, 2017). After activation, A1 astrocytes are involved in neuroinflammation-mediated neurotoxicity in various ways, inducing neuronal and mature differentiated oligodendrocyte death and participating in the progression of neurodegenerative diseases (Liddelow et al., 2017) (Figure 2).

Furthermore, neurodegenerative diseases are distinguished by idiopathic neuronal loss in different parts of the CNS, and these damaged neurons are not compensated by tissue regeneration. Instead, these cells are gradually replaced by extracellular matrix components, which are mainly produced by endothelial cells, activated fibroblasts and astrocytes (D'Ambrosi and Apolloni, 2020). This fibroglial response has dual roles in tissue protection and repair inhibition. Scar-forming astrocytes are usually adjacent to the lesion and prevent the spread of proinflammatory cytokines and cellular debris to some extent (Becerra-Calixto and Cardona-Gómez, 2017). However, a recent study demonstrated that this fibrotic response exacerbates the progression of degenerative diseases. Therefore, it has been suggested that converting the fibrosis-supporting matrix deposition state of astrocytes and myofibroblasts to a fibrosis-supporting regressive or reversible matrix degradation state and reducing scar formation may help to improve the pathological processes of amyotrophic lateral sclerosis (ALS) and AD (D'Ambrosi and Apolloni, 2020).

### Oligodendrocytes

Mature oligodendrocytes (OLs) are found throughout the gray and white matter of the CNS (Boulanger and Messier, 2014). OLs are myelin-forming cells in the CNS and provide metabolic support to neurons by forming myelin sheaths around axons (Saab et al., 2016). In recent years, studies have demonstrated that oligodendrocyte loss and demyelination are characteristic of neurodegenerative diseases. Oligodendrocyte precursor cells (OPCs) are the progenitors of terminally differentiated OLs. As a repair mechanism, when demyelination occurs, the proliferation and differentiation rate of OPCs increases to generate new OLs (Snaidero et al., 2020; Neely et al., 2022). This may be crucial for the pathological recovery process in such diseases. Recent studies have revealed that OLs may also have immune-related functions, with a variety of immunomodulatory factors expressed in OLs, such as cytokines/chemokines and their receptors (Zeis et al., 2016; Raffaele et al., 2021). In multiple sclerosis, OLs and OPCs are not passive targets, but modulators of active immunity (Falcão et al., 2018).

### Endothelial Cells

The neurovascular unit (NVU) establishes close structural and functional connections between neurons, glial cells (astrocytes, oligodendrocytes and microglia) and vascular cells (endothelial cells and pericytes) (Castellani and Schwartz, 2020; Mészáros et al., 2020). NVU contributes to the development and maintenance of the BBB, ion balance, and nutrient transport (Vedam-Mai, 2021). Endothelial cells are an important cellular component of the NVU. Compared to non-neural tissue endothelial cells, brain endothelial cells have high expression of connexins at intercellular junctions, conferring barrier restriction properties for paracellular permeability (Procter et al., 2021). During the inflammatory process, peripheral cytokine act on endothelial cells, leading to impaired barrier function. Meanwhile, inflamed endothelial cells upregulate the expression of adhesion molecules that facilitate the recruitment of circulating peripheral immune cells and antibodies across the barrier (Varatharaj and Galea, 2017; Marogianni et al., 2020). A recent study identified a new pathway for microvascular endothelial cells to degrade myelin debris via the autophagy-lysosome system, which promotes the progression of demyelinating diseases by promoting inflammation, angiogenesis and fibrotic scar formation (Zhou et al., 2019).

### Pericytes

Pericytes are microvascular wall cells that are embedded in the basement membrane and surrounding microvasculature. As mentioned previously, pericytes are important cellular components of the neurovascular unit (Uemura et al., 2020). Pericytes interact with endothelial cells, neuronal cells, glial cells and perivascular macrophages, and this interaction is the basis for performing these functions (Ding et al., 2018; Uemura et al., 2020). In recent years, pericytes have been shown to be involved in neuro-inflammatory as well as neurodegenerative diseases (Nyúl-Tóth et al., 2017). Pericyte-derived pleiotrophin is thought to be a neurotrophic factor required for neuronal survival. The loss of pericytes leads to the breakdown of BBB, blood flow and loss of neurons in the mouse brain (Nikolakopoulou et al., 2019).

## The Peripheral Immune System

### Neutrophils

During infection, neutrophils are recruited to the area of infection to engulf invading microorganisms through phagocytosis. Under physiological conditions, there are almost no neutrophils present in the CNS due to the BBB (Guo et al., 2021). However, under different pathological conditions, such as infection, trauma or neurodegeneration, a large amount of neutrophil infiltration occurs in the CNS.

Studies have shown that neutrophils disrupt the BBB by releasing free radicals, proteolytic enzymes and matrix metalloproteinases (MMPs) (Strecker et al., 2017; Manda-Handzlik and Demkow, 2019). Neutrophil extracellular traps (NETs) are reticulated ultrastructures that are released into the extracellular space after neutrophil activation and damage the BBB (Naegele et al., 2012). Zenaro et al. observed neutrophil-microglia crosstalk in AD and the presence of NETs within blood vessels and the brain parenchyma, suggesting that NETs affect the BBB in Alzheimer's disease and contribute to neuronal damage (Zenaro et al., 2015). In addition, the authors showed that the migration of neutrophils produces IL-17 in the cortex and hippocampus, which not only has a direct toxic effect on neurons and is involved in the disruption of the BBB but recruits additional neutrophils (Kebir et al., 2007; Zenaro et al., 2015). Disruption of the BBB has become one of the mechanisms of peripheral-central nervous system crosstalk and is an early marker of neurodegenerative diseases (Giannoni et al., 2020; Ishii and Iadecola, 2020).

### Monocytes

Monocytes are the largest white blood cells in peripheral blood and can differentiate into monocyte-derived macrophages (MDMs) or monocyte-derived dendritic cells (moDCs). Similar to neutrophils, monocytes are barely detectable in the CNS under physiological conditions (Gomez Perdiguero et al., 2015; Mrdjen et al., 2018; Croese et al., 2021). When brain homeostasis is disrupted, MDMs can invade the brain through an intact or compromised BBB (Engelhardt et al., 2017; Greenhalgh et al., 2020). Infiltrating blood-derived monocytes play a major role in controlling neuropathic events in the CNS, including scar degradation, anti-inflammatory and neurotrophic factor production (Shechter et al., 2013; Schwartz, 2017). Greenhalgh et al. (2018) found evidence of direct communication between MDMs and microglia and differential regulation of each other's functions. MDMs inhibit the critical functions of microglia and counteract harmful acute and long-term microglia-mediated inflammation.

### NK Cells

NK cells are bone marrow (BM)-derived haematopoietic cells that are widely distributed in peripheral lymphoid organs and the circulatory system (Hazenberg and Spits, 2014). NK cells can secrete cytokines and chemokines that affect the host immune response or release cytoplasmic granules containing perforin and granzyme to induce apoptosis in target cells (Voskoboinik et al., 2015; Zhou et al., 2020). In addition, some NK cells can kill target cells through the death receptor pathway via Fas ligand and tumor necrosis factor-related apoptosis-inducing ligand (TRAIL) (Guillerey et al., 2016; Yang et al., 2018). Under pathological conditions, NK cells are recruited to the CNS by chemokines. For example, during neuroinflammation, neuronal production of CX3C chemokine ligand 1 (CX3CL1) is necessary to direct CX3CR1-expressing NK cells into the brain (Huang et al., 2006; Hamann et al., 2011).

NK cells have different immunomodulatory functions in different neurological diseases. For example, NK cells interact with glial cells to regulate the neuroinflammatory response in neurodegenerative diseases (Huang et al., 2006; Hertwig et al., 2016). NK cells regulate Treg recruitment and microglial phenotype by interacting with infiltrating Treg cells and resident microglia. In addition, NK cells affect CNS physiology by killing glial cells and secreting IFN-γ (Saikali et al., 2007; Moodley et al., 2011).

### Dendritic Cells (DCs)

Dendritic cells (DCs) are the predominant antigen-presenting cells (APCs). In the steady state, DCs are present in the CNS, expressing major histocompatibility complex class II (MHCII) and the leukocyte integrin CD11c (Santos et al., 2020). The expression of MHCII and CD11c is generally used to identify the existence and position of DCs in the brain (Bulloch et al., 2008; Colton, 2013). Through the identification of cell morphology and surface markers, multiple studies have shown that under physiological conditions, DCs are located in the meninges, choroid plexus, cerebrospinal fluid, and perivascular spaces in the CNS (McMenamin et al., 2003; Schain et al., 2018). These sites are all highly vascularized regions, supporting the idea that cerebral DCs are of vascular origin rather than from within the brain. Anandasabapathy et al. (2011) demonstrated that endogenous dendritic cells in the meninges and choroidal plexus of the steady-state brain are most likely derived from local precursor dendritic cells that entered the perivascular region of the brain early in life, and this conclusion suggests that such precursor dendritic cells are derived from bone marrow rather than monocytes.

The release of chemokines and adhesion molecules allows peripheral DCs to migrate to the meninges or choroid plexus, recognize antigens and present antigens to T cells (Sabahi et al., 2021). DCs can provide the co-stimulatory signals required for T cell activation, promoting the activation and proliferation of CD8+ cytotoxic T cells (CTLs) and CD4+ helper T cells with diverse proinflammatory cytokine profiles (Colton, 2013). These proinflammatory effects may cause tissue damage under certain conditions. However, a recent study showed that the bone marrow-derived dendritic cells induce neuroprotective Tregs in a PD model, providing neuroprotective effects through modulation of adaptive immunity (Schutt et al., 2018). In conclusion, these studies demonstrate the role of DC in the regulation of inflammatory and neurodegenerative diseases.

### T Cells

T cells are key immune cells of the adaptive immune system. Several studies have shown that the connection between the innate and adaptive immune systems is also implicated in the progression of neurodegenerative diseases. T cells within the CNS are often considered pathogenic, especially in the context of neuroinflammatory diseases, and excessive inflammatory responses are thought to be modulators of the pathogenesis of neurodegenerative diseases (Ellwardt et al., 2016). Abnormal T cells promote neuroinflammation through direct crosstalk with glial cells in the brain and the secretion of pro-inflammatory mediators (Dai and Shen, 2021). However, recent studies have shown that T cells play an active role in limiting inflammation and CNS damage, infection and neurodegeneration (Ellwardt et al., 2016). The balance between T cells, which can play an injurious or protective role in CNS, remains to be further investigated.

CD4+ T cells are the main regulators of the immune response by secreting a variety of cytokines, recruiting immune cells to the site of infection, and initiating the differentiation of CD4+ T cell subsets with different effector functions (Morgan et al., 2021). Naïve CD4+ T cells (Th0) differentiate into antigen-specific T effector cells, including T helper 1 (Th1), T helper 2 (Th2), and T helper 17 (Th17) cells and regulatory T cells (Tregs) (Figure 2). CD8+ T cells mainly mediate cellular immune responses and are known as cytotoxic T cells (CTLs).

Th1 and Th17 cells are generally regarded to be producers of pro-inflammatory cytokines and directly promote neuroinflammation by secreting inflammatory mediators such as IL-1, IL-6, IL-17, TNF-α and IFN-γ (Dardalhon et al., 2008). Furthermore, these cells enhance microglia-mediated neurotoxicity by upregulating the release of reactive oxygen species (ROS) and nitric oxide (NO) (Liu et al., 2020). In contrast, Th2 cells are thought to be anti-inflammatory and produce anti-inflammatory cytokines (IL-4). Th2 cells and Tregs enhance microglia-mediated neuroprotection (Gendelman and Appel, 2011; Mayne et al., 2020). Th1 and Th2 cells are essential for maintaining a healthy CNS environment, the ratio of Th1 to Th2 cytokines (Th1/Th2) can be used to reflect the pattern of the immune response, and alterations in this ratio are thought to be a trigger for neurodegenerative diseases (Burgaletto et al., 2020).

The critical role of regulatory T lymphocytes (Tregs) in immune tolerance and the control of inflammatory responses makes them potential therapeutic targets for many diseases (Sheean et al., 2018). Tregs exert neuroprotective effects by regulating microglial activation. In the early stages of ALS, this regulation manifests as the upregulation of Tregs and elevated levels of M2-type neuroprotective microglia (Beers et al., 2011). However, as the disease progresses, Treg levels decrease, pro-inflammatory cytokine levels increase, and microglia shift to a neurotoxic M1 phenotype (Chen et al., 2014; Sheean et al., 2018). Studies have shown that the number of Tregs is negatively correlated with the progression of ALS (Beers et al., 2011). In SOD1-mutant mice, passive transfer of mSOD1 Tregs to ALS mice lacking functional T lymphocytes induced M2 microglia in the spinal cord and prolonged survival (Banerjee et al., 2008; Beers et al., 2011).

In humans, gamma/delta (γδ) T cells are a relatively small subset of T lymphocytes (Xu et al., 2020). γδ T cells function as innate immune cells, and they have recently been shown to share many key features of adaptive immunity (Davey et al., 2017, 2018). Previous studies have demonstrated that γδ T cells can regulate immune responses associated with inflammation and have a pro-inflammatory role in the CNS. γδ T cells can promote local amplification of the immune response in the CNS, altering the interstitial microenvironment of the inflamed brain and ultimately leading to BBB disorders (Schirmer et al., 2013; Benakis et al., 2016; Wo et al., 2020). However, Ponomarev et al. showed that γδ T cells could regulate CNS inflammation and disease recovery through Fas/Fas ligand-induced brain-native T cell apoptosis (Ponomarev and Dittel, 2005). The different functions depend on the time and location of γδ T cells, as well as on the different subtypes. However, more evidence on different subtypes of γδ T cells is needed to determine their various roles in CNS inflammation (Wo et al., 2020).

CD8+ T cells have been shown to be involved in the pathophysiology of diseases associated with neurodegeneration. CD8+ T cells drive CNS axonal degeneration in normal senescent mice in a T cell receptor- and granzyme B-dependent manner, and this deleterious effect is further enhanced in the presence of inflammation (Groh et al., 2021). In addition, Coque et al. (2019) found that autoreactive CD8+ T cells directly interacted with motor neurons and triggered death. Recently, however, there has been an increase in interest in CD8+ T cells, as CD8+ T cells have been shown to have other functions than neurotoxicity. Studies have shown that peripheral infection induces a type of CNS tissue-resident memory CD8+ T cells in the brain. These memory CD8+ T cells contribute to the control of CNS infection, showing rapid activation, enhanced cytokine production and mediated protection after brain infection (Griffin and Metcalf, 2011; Urban et al., 2020).

### B Cells

B cells are important regulators of CNS homeostasis and disease states and play an important role in the pathogenesis of various CNS diseases by acting peripherally or compartmentalizing within the CNS. In individuals with CNS inflammation, the number of B cells in the cerebrospinal fluid increased several-fold, and in the CNS parenchyma and perivascular space, these cells increased at least several orders of magnitude (Machado-Santos et al., 2018).

In some PD patients, decreased levels of B lymphocyte subsets in the peripheral blood were detected, which may be related to altered B cell-related gene expression (Kobo et al., 2016). Although no B cells have been identified in the post-mortem brain tissue of PD patients, studies have shown IgG deposition on dopaminergic neurons in the substantia nigra and Lewy bodies in the CNS (Orr et al., 2005). Another study showed increased levels of alpha-synuclein-specific autoantibodies in the blood and cerebrospinal fluid of PD patients (Shalash et al., 2017). This finding reflects the role of B cell antibodies in PD. Despite the mounting evidence of the involvement of B cells in neurodegenerative diseases, whether the changes in adaptive immune are causal or secondary to CNS injury still needs further study (Sabatino et al., 2019).

## Regulation of Peripheral and Central Immunity in Neurodegenerative Diseases

### Amyotrophic Lateral Sclerosis (ALS)

Amyotrophic lateral sclerosis (ALS) is a severe degenerative disease of the CNS. ALS is characterized by the progressive degeneration of upper motor neurons in the motor cortex and lower motor neurons in the brainstem and spinal cord, resulting in severely impaired motor function in patients (Brown and Al-Chalabi, 2017). The main symptoms of ALS are related to motor dysfunction, and patients experience progressive muscle weakness. As the disease progresses, patients gradually lose all muscle control, eventually leading to death, with an average life expectancy of only 2-5 years after onset (Tunca et al., 2018). An increasing number of studies have shown that patients with ALS can develop non-motor symptoms, such as cognitive and behavioral impairments during the course of the disease in 50% of patients and concomitant behavioral variant frontotemporal dementia (FTD) in 13% of patients (Elamin et al., 2013; Hardiman et al., 2017). Most ALS cases are sporadic (SALS) with no clear genetic origin. Only approximately 10% of cases are familial ALS (FALS) caused by genetic mutations, of which Cu^2+^/Zn^2+^ superoxide dismutase (SOD1) mutations are considered to be the most prominent and earliest identified genetic cause of ALS. Other disease-specific gene mutations include the C9orf72, TDP43, and FUS mutations (Sreedharan et al., 2008; Vance et al., 2009; Renton et al., 2011).

Neuroinflammation is thought to be involved in the heterogeneity of ALS. The crosstalk between activated microglia and astrocytes and pro-inflammatory peripheral immune cells in the CNS, together with the immune molecules these cells release, is significantly associated with disease progression and survival in ALS patients. We summarized the emerging role of the peripheral and central immune responses in ALS with the aim of providing clear insights into potential new treatments for ALS.

Microglia are known to play dual roles in the pathogenesis of ALS. It has been shown that more than 90% of ALS patients have accumulated cytoplasmic TDP-43 aggregates in the post-mortem spinal cord (SC) (Spiller et al., 2016). TDP-43 increases BBB permeability and impairs the release of neurovascular unit components, leaving the brain vulnerable to systemic immune responses during inflammation (Zamudio et al., 2020; de Boer et al., 2021). The novel inducible mouse rNLS8 model of ALS was used to show that activated microglia selectively cleared neuronal hTDP-43, exerting an important neuroprotective effect (Spiller et al., 2018). Furthermore, Liao et al. observed increased mRNA levels of the M2 phenotype markers Ym1, CD163 and BDNF in murine mSOD1 microglia during the early stages of ALS, suggesting that microglia express the M2 anti-inflammatory phenotype during the early stages of ALS (Liao et al., 2012). However, during ALS disease progression, mSOD1-expressing microglia have dual phenotype and functional profiles. As the disease progresses, mSOD1 microglia in the spinal cords of ALS mice have increased levels of the M1 marker NOX2 and secrete reactive oxygen species (ROS) and pro-inflammatory cytokines (Frakes et al., 2014; Zhao et al., 2015). Frakes et al. (2014) demonstrated that NF-κB was activated in the G93A mouse model as ALS progressed and regulated the conversion of ALS microglia to a pro-inflammatory, neurotoxic state.

Studies of human cases and animal models of ALS have shown that astrocytes are a key factor in the progression of ALS. On the one hand, astrocytes play a role in the specific degeneration of spinal cord motor neurons in ALS. On the other hand, astrocytes can regulate BBB permeability and can crosstalk with CNS immune components and infiltrating peripheral immune components (Novellino et al., 2020). A recent study showed that transforming growth factor-β1 (TGF-β1) expression was upregulated in astrocytes in murine and human ALS (Endo et al., 2015). TGF-β1 is thought to be a negative regulator of the neuroprotective inflammatory response, forming an IFN-γ-dominated environment in infiltrating T cells. The overproduction of TGF-β1 hastens disease progression by disrupting the protective effects of microglia and T cells (Endo et al., 2015). In addition, previous studies have demonstrated that astrocytes can amplify the dual effects of microglia during the pre-symptomatic and symptomatic phases. In the SOD1^G93A^ ALS model, IKK2/NF-κB activation in astrocytes drives Wnt-dependent microglial proliferation (Ouali Alami et al., 2018). This response prolongs the pre-symptomatic phase of ALS, delays muscle denervation, and reduces disease burden. However, during the symptomatic phase, this response enhances pro-inflammatory microglial activation, leading to accelerated ALS progression and shortened survival (Ouali Alami et al., 2018). Therefore, enhancing pre-symptomatic immune responses may be a viable therapeutic option for pre-symptomatic patients.

Mast cells are associated with myofibers and motor endplates and form NETs by interacting with neutrophils (Trias et al., 2018). Mast cells, phagocytic neutrophils, and NETs were abundant around the neuromuscular junction (NMJ) and in degenerating motor axons, which that suggested a role for immune effector cell interactions in driving ALS progression (Trias et al., 2017, 2018).

The topic of whether monocytes infiltrate the CNS and their impact on ALS progression is currently controversial. A parabiosis experiment found that CNS microglia are a closed system with no evidence of recruitment from the circulation (Ajami et al., 2007). However, another study showed that in the glial cells of ALS patients, there is increased expression of the chemokine MCP-1, which attracts monocytes and myeloid dendritic cells, which in turn recruit monocytes and moDCs (Henkel et al., 2004). Furthermore, Zondler et al. (2016) demonstrated that in ALS, circulating monocytes are dysregulated in terms of both subtype composition and function and that peripheral monocytes invasion of the CNS is increased. To some extent, peripheral monocytes have a protective role during the early stages of ALS. In a recent study, Komiya et al. assessed whether CCR2+ monocytes infiltrated the CNS of ALS mice by examining the distribution of the CCR2 protein in a CCR2-reporter mouse model (Komiya et al., 2020). This study demonstrated that during ALS disease progression, CCR2 expression expanded from CNS-infiltrating monocytes to centrally resident microglia and neurons, resulting in the toxic transformation of microglia and neurons, leading to accelerated ALS pathology.

As mentioned above, despite the controversy, a growing number of studies have shown infiltration of peripheral monocytes into the CNS. However, it is controversial whether infiltrating monocytes have a beneficial or detrimental effect on ALS progression. Therefore, future studies on monocytes are needed to provide new insights into the pathogenesis of ALS.

In ALS, NK cells determine the onset and progression of motor neuron degeneration. Garofalo et al. (2020) found that in hSOD1^G93A^ mice, NK cells can directly kill spinal cord motor neurons. In addition, NK cells can also interact with resident microglia and infiltrating Treg cells, contributing to the motor impairment. A recent study demonstrated that NK cells promote ALS progression in a gender- and age-specific manner, which suggests that we should consider gender and age variables when designing immunotherapy for ALS (Murdock et al., 2021).

In addition to the innate immune response, the adaptive immune system is involved in the pathological process of ALS. In mSOD1 transgenic mice, CD4+ T cells provided supportive neuroprotection by modulating the trophic and cytotoxic balance of glial cells (Beers et al., 2008). Among them, Tregs act as suppressors of the excessive immune responses. In mice with a mutant form of SOD1, secondary transfer of Tregs induced the M2 phenotype in microglia in the spinal cord and delayed the onset of clinical symptoms, demonstrating the neuroprotective effect of Tregs by modulating the activation of microglia (Banerjee et al., 2008; Sheean et al., 2018). Tregs tend to increase in the early stages of ALS, suppressing microglial activation by secreting IL-4 (Beers et al., 2017). As the disease progresses, the increased levels of proinflammatory cytokines lead to the transformation of microglia into a neurotoxic M1 phenotype, which may be associated with a decreasing tendency of Tregs due to the absence of FoxP3 expression (Beers et al., 2017). During the rapid developmental stage of ALS, not only do Tregs levels decrease, but patients show an increase in Th1 and Th17 proinflammatory T cell subsets and a decrease in Th2 cells in peripheral blood, and the immune phenotype is skewed toward a Th1/Th17 cell-mediated proinflammatory phenotype that correlates with the severity and progression of the disease (Jin et al., 2020).

In contrast, CTL infiltration in the CNS of ALS patients and mSOD1 mice is usually considered detrimental to motor neurons. MHCI is a key molecule associated with the interaction of monocytes with CD8+ T lymphocytes. Coque et al. showed that SOD1^G93A^-expressing CD8+ T cells selectively triggered motor neuron death in an MHCI-dependent manner via the granzyme and Fas death pathways (Coque et al., 2019). In addition, Nardo et al. showed that CD8+ T cells interacted with microglia expressing MHCI, accelerated motor neuron death and reduced survival in SOD1^G93A^ mice (Nardo et al., 2018). However, CD8+ T cells were shown to be more than just neurotoxic. Sustained expression of MHCI in motor neurons protected mSOD1 mice from ALS astrocyte-induced toxic effects (Song et al., 2016). Nardo et al. (2018) showed that the activation of MHCI in the peripheral nervous system of ALS mice was considered an early protective response. In addition, damaged MNs in SOD1-associated ALS actively recruit immune cells. Infiltration of CD8+ T cells and macrophages promotes myelin regeneration, delays muscle denervation and prolongs survival time (Nardo et al., 2016).

In summary, the role of the immune system is often complex and multifaceted. The same cell groups will have a positive or negative effect at different stages of the disease or with different stimuli. Future studies could focus on these different stages and the modulation of stimuli to promote more neuroprotective effects of immune cells.

### Alzheimer's Disease (AD)

Alzheimer's disease (AD) is the most common neurodegenerative disease and the most common cause of cognitive decline in the elderly population. Characteristic pathological changes in AD include the deposition of β-amyloid (Aβ) in the brain to form senile plaques and the hyper-phosphorylation of tau protein to cause neurofibrillary tangles (NFTs) (Chen and Mobley, 2019). Despite tremendous efforts to determine the etiology of AD, the exact pathogenesis of AD is not fully understood, and there are no effective drugs or therapies to stop or reverse the progression of AD. Numerous studies have demonstrated that neuroinflammation plays a key role in the pathogenesis of AD and that crosstalk between peripheral immune cells and the CNS is involved in the onset and progression of AD. To date, the role of peripheral immunity in AD is still not well understood, although a large number of studies have demonstrated its involvement in all phases of AD.

Microglia may have dual roles in the pathogenesis of AD (Li et al., 2020). Activated microglia have the capacity to remove excess Aβ plaques and cellular debris (Ries and Sastre, 2016). Loss-of-function mutations in genes associated with microglial transmembrane proteins, such as TREM2 and CD33, are associated with reduced microglial phagocytosis and are considered genetic risk factors for AD (Hansen et al., 2018; Cao et al., 2021). As the disease progresses, the function of microglia changes. On the one hand, continuous stimulation with high cytokine concentrations leads to the transformation of microglia into a dysfunctional senescent state, with diminished phagocytosis leading to Aβ accumulation and the loss of neural support functions (Hickman et al., 2008; Salani et al., 2019). One the other hand, the danger-associated molecular patterns (DAMPs), including Aβ and phosphorylated tau, are the activators of microglia. These persistent stimuli drive chronic microglial activation, making them neurotoxic (Thadathil et al., 2021). Astrocytes can take up and internalize Aβ from the extracellular environment and participate in its degradation. A recent study demonstrated that IL-3 derived from astrocytes in the mouse brain was protective in a mouse model of AD and served as a key mediator of astrocyte-microglia crosstalk, which may be a strategy for therapeutic intervention in AD (McAlpine et al., 2021). However, the pathological role of reactive astrocytes in AD has been demonstrated. Chun et al. (2020) found that heavily reactive astrocytes could cause neurodegeneration in AD and act as a new hallmark of AD. This damage may be due to hydrogen peroxide production by heavily reactive astrocytes leading to neuronal death, brain atrophy, and cognitive impairment. Aβ plaques and tau aggregates can stimulate NLRP3 inflammasome within astrocytes and microglia cells, thereby causing activation of caspase-1 and the release of inflammatory cytokines that trigger pathophysiological changes in AD (Haseeb et al., 2022). The inhibition of NLRP3 inflammasome reduces pathological features such as Aβ deposition and Tau phosphorylation.

In addition, pericytes of the CNS are involved in AD pathology. In a mouse model of AD, pericytes are involved in Aβ-induced capillary constriction in the brain (Nortley et al., 2019). The loss of pericytes accelerates amyloid angiopathy and brain amyloidosis by reducing the clearance of Aβ from the interstitial fluid of the brain (Sagare et al., 2013).

Monocytes appear to have dual roles in AD pathophysiology. Murine monocytes were reorganized into two major subpopulations based on their chemokine receptor and Ly6C expression levels: a pro-inflammatory subset and an anti-inflammatory subset. On the one hand, the Ly6C^*low*^ monocyte subpopulation patrolled the vascular lumen and enhanced tissue repair, phagocytosed toxic elements, including Aβ, and alleviated neurodegenerative processes (Naert and Rivest, 2013). On the other hand, Ly6C^*high*^ monocytes could infiltrate the brain parenchyma and produce pro-inflammatory cytokines that promoted microglial activation (Fani Maleki and Rivest, 2019).

A recent study demonstrated that the inflammatory properties of circulating neutrophils change with increasing age and that neutrophil phenotype may correlate with the rate of cognitive decline in AD patients. Thus, an altered neutrophil phenotype may serve as a prognostic blood biomarker for AD disease progression (Dong et al., 2018). In addition, the results of animal studies of AD suggest that neutrophils may be associated with the destruction of the BBB (Baik et al., 2014). Zenaro et al. (2015) observed in transgenic model mice with AD that the integrin LFA-1 controls neutrophil extravasation into the CNS and parenchyma and is present in areas with Aβ deposits. Neutrophils in these areas are directly toxic to neurons and the BBB by releasing NETs and IL-17 and may recruit more neutrophils into the CNS (Kolaczkowska and Kubes, 2013). Neutrophil depletion or integrin LFA-1 blockade reduced AD severity in animal models, suggesting that neutrophil-directed therapy may benefit AD patients (Zenaro et al., 2015).

Infiltration of the brain by peripheral NK cells and the resulting neuroinflammatory changes have been observed in human AD and 3xTg-AD mice (Zhang et al., 2020; Lu et al., 2021). It has been suggested that NK cells are biomarkers of the early stages of AD (Le Page et al., 2015, 2018). A study showed that the accumulation of NK cells in the aging brain impairs neurogenesis, and NK cell depletion reduces neurogenesis and neuroinflammation in the aging brain and AD patients (Jin et al., 2021). Another study demonstrated the critical role of NK cells in promoting neuroinflammation and AD-related cognitive decline. Depletion of NK cells with an anti-NK1.1 antibody significantly improved cognitive function in 3xTg-AD mice, and microglia from NK cell-depleted 3xTg-AD mice exhibited homeostatic-like morphology and decreased expression of pro-inflammatory cytokines (Zhang et al., 2020). In summary, targeting NK cells and neuroinflammation may provide new pathways for the treatment of AD. NK cell killing and degranulation remain unchanged during healthy aging and AD development, although numerous changes in NK phenotype and function occur (Le Page et al., 2015). Further studies on the altered phenotype and function of NK cells may help to provide insight into the relationship between NK cells and aging or with neurodegenerative diseases.

In contrast, the role of adaptive immunity in AD has not been adequately explored. Increased Aβ-specific CD4+ T cell and B cell responses were found in the blood samples of AD patients, suggesting that Aβ can antigenically induce adaptive immune responses (Monsonego et al., 2003). In a Rag-5 × fAD mouse model of T-, B- and NK cell-deficient mice, the Aβ plaque load was significantly increased, the neuroinflammatory phenotype of microglia was exacerbated, and phagocytosis was reduced (Marsh et al., 2016). This finding suggests that the adaptive immune system plays an important role in limiting AD amyloid pathology. However, Kim et al. observed the progression of AD required B cells (Kim et al., 2021). At the onset of the disease, therapeutic depletion of B cells significantly delayed the progression of AD in mice. Meanwhile, in another study, decreased brain Aβ levels and increased microglial proliferation were observed in an aged PSAPP mouse model of functional T and B cell ablation (Späni et al., 2015). These conflicting findings suggest that the role of the adaptive immune system in the development of AD remains controversial.

The role of Tregs in AD pathophysiology has been controversial in recent years. The transplantation of Treg cells into 3xTg-AD transgenic mice led to the observation of improved AD pathology in mice, as well as the observation of reduced brain Aβ load and reduced production of inflammatory cytokines (Baek et al., 2016). However another study found that a transient depletion of Foxp3(+) Tregs may contribute to AD disease remission (Baruch et al., 2015). Such conflicting results imply a complex role for Tregs in AD pathology. It is worth noting that these two conflicting results may be due to the different disease stages. In the early stages of the disease, Tregs may promote beneficial activation of microglia and inhibit deleterious pro-inflammatory glial proliferation (Dansokho et al., 2016). However, in later stages of the disease, systemic Foxp3+ Treg plays a negative role in the pathology of AD by altering the function of the choroid plexus and thereby reducing leukocyte recruitment to the CNS (Baruch et al., 2015). Future studies are needed to evaluate the therapeutic potential of Treg-based immunomodulatory approaches in AD.

Due to the altered permeability of the BBB in AD patients, immune cells can travel to and from the brain. Recent studies have shown that crosstalk between microglia and astrocytes is critical for T cell recruitment to the CNS (Burgaletto et al., 2020). Previous studies have demonstrated an increased percentage of lymphocytes in the brain parenchyma of AD patients. A recent study demonstrated the presence of clonally expanded CD8+ TEMRA cells in the cerebrospinal fluid of AD patients and that clonally expanded CD8+ T cells patrolled the cerebrospinal fluid during age-related neurodegeneration (Gate et al., 2020). Laurent et al. demonstrated the activation of microglia and astrocytes in a THY-Tau22 mouse model of tau pathology and cognitive dysfunction (Laurent et al., 2017). Furthermore, the infiltration of CD8+ T cells associated with early chemokine responses, particularly those involving CCL3, was observed in the hippocampus in mice. This finding demonstrates the important role of adaptive immunity in AD pathophysiology.

### Parkinson's Disease (PD)

Parkinson's disease (PD), which is also known as tremor palsy, is the second most common neurodegenerative disease after AD. PD is characterized by a prominent loss of dopaminergic neurons in the substantia nigra (SN) and pathological intraneuronal aggregation of alpha-synuclein (α-syn) in Lewy vesicles (Shahnawaz et al., 2020). PD pathogenesis is still unclear, and the increased incidence is related not only to aging but to environmental factors and genetic defects that can lead to the degeneration of dopaminergic (DA) neurons in the brain. Leucine-rich repeat kinase 2 (LRRK2) is the most commonly mutated gene in familial PD (Deniston et al., 2020). Over the past decade, an increasing number of studies have focused on the role of the immune system in AD, and pro-inflammatory immune-mediated mechanisms are believed to play important roles in disease progression. However, the extent to which changes in peripheral immunity affect the CNS remains a matter of debate.

Since the discovery of activated microglia in the substantia nigra pars compact (SNpc) of the midbrain in PD patients by McGeer et al. (1988), microglial activation and subsequent neuroinflammation have been shown to play multiple roles in the degeneration of dopaminergic neurons in AD patients. Microglia contribute to the clearance of misfolded α-syn aggregates in PD (Brück et al., 2016). However, α-syn can in turn activate NLRP3 inflammasome in microglia through interaction with Toll-like receptors (TLR). This leads to translocation of NF-κB, which induces increased expression of pro-inflammatory cytokines as well as impaired mitochondria, thereby damaging dopaminergic neurons (Gustot et al., 2015). Targeting the α-syn/TLRs/NF-κB/NLRP3 inflammasome axis may have some potential application in the treatment of PD (Li et al., 2021). However, at present, modulators of inflammasome are limited by clinical effectiveness as well as safety factors, making it difficult to achieve translation to the clinic.

In addition, LRRK2 mutations associated with PD can drive microglial activation, leading to increased microglial phagocytosis and increased production of inflammatory factors, as well as reactive oxygen species (ROS) (Subramaniam and Federoff, 2017; Kim et al., 2018). Microglia, which were originally neuroprotective, become toxic to dopaminergic neurons due to the overproduction of cytokines and ROS. In addition to their recognized role in neuroinflammation, glial cells are involved in the intercellular transmission of α-syn through exosome release. Guo et al. (2020) observed that exosomes released from microglia could induce nigrostriatal degeneration and play a key role in the pathogenesis of PD.

Immunoreactive astrocytes with elevated density and phenotypic changes have been identified in post-mortem PD brains; however, the specific function of astrocytes in PD pathology remains unclear (Braak et al., 2007). On the one hand, astrocytes can play a protective role in disease progression by effectively isolating and degrading pro-inflammatory extracellular α-syn. On the other hand, high concentrations of extracellular α-syn induce astrocyte inflammatory responses in a TLR4-dependent manner, which may exacerbate stressful conditions in brains with synucleinopathy (Rannikko et al., 2015). A recent study by Sonninen et al. (2020) suggested that LRRK2- and GBA-mutant astrocytes contributed to the development of PD. In addition, the activation of microglia by classic inflammatory mediators converts astrocytes to the neurotoxic A1 phenotype. Yun et al. (2018) found that NLY01 was a potent GLP1R agonist with good neuroprotective effects by directly blocking the microglia-mediated conversion of astrocytes to the A1 neurotoxic phenotype. Astrocytes are involved in the disruption of the BBB in PD patients. Lan et al. (2022) found that α-syn oligomers lead to the activation of astrocytes, which increased the production and release of vascular endothelial growth factor A (VEGFA) and nitric oxide (NO), both of which can lead to the degradation of BBB integrity. It is worth noting that recent studies have demonstrated that PD is also associated with OLs (Bryois et al., 2020). Agarwal et al. (2020) described the human single-nuclei transcriptomic atlas for the SN and found a significant association between the risk of PD and oligodendrocyte-specific gene expression, which also reveals a possible role of OLs in the etiology of PD.

Monocytes may be an essential element in the pathogenesis of PD, and the overexpression of expressed quantitative trait loci (eQTL), which is specific to monocytes, has been shown to be associated with PD (Raj et al., 2014). The disease-specific gene expression profile of peripheral blood mononuclear cells in early AD correlates with the severity of the disease (Schlachetzki et al., 2018). Grozdanov et al. (2014) demonstrated the dysregulation of peripheral blood monocytes in PD patients, in which an increased proportion of pro-inflammatory monocytes was accompanied by activation of the CCR2-CCL2 axis in PD. The authors suggest that this increase in classic monocytes and elevated CCL2 serum levels may be related to the secretion of inflammatory mediators by microglia. The FAS/FASLG system, which regulates monocyte subpopulations, may be a potential target for PD therapy. A recent study showed that pathological α-syn activates LRRK2 expression and kinase activity in monocytes, promoting the recruitment of pro-inflammatory monocytes to the brain, which in turn drives the neuroinflammatory response in PD. Thus, LRRK2 kinase inhibitors may attenuate pro-inflammatory monocyte responses in the brain (Xu et al., 2022).

The role of NK cells in PD pathogenesis is still unclear. However, a recent study showed that NK cells have a protective effect on Lewy body (LB)-related neurodegenerative diseases. On the one hand, human NK cells can efficiently internalize and degrade α-syn aggregates via the endosomal/lysosomal pathway. On the other hand, NK cells are involved in resolving extracellular α-syn load by producing IFN-γ and activating or differentiating antigen-presenting cells, including microglia. In a preclinical mouse model of PD, systemic depletion of NK cells led to worsened motor symptoms and nuclear protein pathology (Earls et al., 2020). Recent evidence indicated that the misfolded a-syn may be retrogradely transported from the enteric nervous system to the CNS along the vagus nerve (Hill et al., 2021). Therefore, whether immune cells affect α-syn pathology in the periphery, especially in the intestine, may be an important research direction for future studies.

There is increasing consensus that the adaptive immune system is also involved in the pathogenesis of PD (Tansey and Romero-Ramos, 2019). CD4+ T cells and CD8+ T cells have been found in the SN region in postmortem specimens from PD patients and MPTP-induced mice, and this immune response promotes dopaminergic neuron (DN) degeneration through the Fas/FasL cytotoxic pathway (Brochard et al., 2009). Sommer et al. found that a large amount of Th17 is present in the substantia nigra of PD patients and that IL-17 secreted by T lymphocytes is essential for neuronal death (Sommer et al., 2018). Additionally, in an *in vitro* model constructed using patient-induced multifunctional stem cells, antagonism of both IL-17 and its receptor was able to prevent neuronal death. In addition, the adaptive immune system interacts with immune cells in the CNS. It is suggested that glial cells may be involved in the Th17-mediated cell death of PD neurons described above (Muffat et al., 2016; Sommer et al., 2018). Activated microglia secrete inflammatory mediators that mediate antigen presentation to CD4+ T cells via the MHC-II pathway, resulting in cell proliferation, slow degeneration and dopaminergic neuronal death (Marogianni et al., 2020).

However, Tregs are a subtype of CD4+ T cells. Tregs exert neuroprotective effects on animal models of PD by inhibiting immune activation and microglial attack of α-syn and preventing the loss of dopaminergic neurons in the substantia nigra (Reynolds et al., 2007). Recent studies have shown that the pathogenesis of PD involves two stages of CTL-mediated immune responses. The first stage is early robust CTL infiltration, which causes slight neuronal loss and α-syn aggregation, but no dopaminergic neuronal death was found at this stage. More modest CD8+ T cell infiltration was observed in the next stage, amplifying α-synuclein pathology and neurodegeneration. The authors suggested that CD8+ T cells promote substantia nigra dopaminergic neuron dysfunction and death in PD prior to the appearance of overt Lewy bodies (Galiano-Landeira et al., 2020). This pathogenicity of CD8+ T cells should be confirmed by appropriate models. Further studies on the number and phenotype of infiltrating CTL at different stages of the disease could help in the development of immunotherapies targeting these T cells.

In summary, we have presented the emerging role of the central and peripheral nervous systems in ALS, AD and PD. Meanwhile, the immune system also plays a crucial role in other neurodegenerative diseases, such as multiple sclerosis or Huntington's disease.

Multiple sclerosis (MS) is traditionally defined as a chronic immune-mediated demyelinating disease of the CNS. The activation of glial cells plays a key role in the process of demyelination, neuronal and axonal damage (Baaklini et al., 2019). Similar to the pathogenesis of AD, neuroinflammation in MS is also characterized by the activation of microglia and astrocytes in the CNS. This leads to the secretion of additional pro-inflammatory cytokines and chemokines that further exacerbate neuroinflammation and BBB destruction, thereby recruiting more peripheral immune cells (Linnerbauer et al., 2020; Vainchtein and Molofsky, 2020). In addition, such interactions between glial cells have an effect on OPC/OLs, thus affecting the demyelination remyelination process (Chu et al., 2021).

Approximately 85% of patients present with a relapsing-remitting phenotype, which is dominated by peripheral immune responses. Peripheral immune cells disrupt BBB infiltration into the brain parenchyma, producing focal areas of primary demyelination (Lassmann, 2018). In contrast, the primary and secondary progressive forms of MS are dominated by neurodegeneration and enhanced innate immune responses, resulting in severe axonal damage, neuronal death and synaptic loss (Faissner et al., 2019). In contrast to other neurodegenerative diseases, immunotherapies have had great success in targeting relapsing-remitting MS. These therapies primarily target the peripheral immune system and therefore have limited effectiveness in the treatment of progressive MS (Healy et al., 2022). Further studies on the activation state, pathogenic role and interaction of glial cells with peripheral immune cells may help to identify the new potential therapeutic opportunities.

Huntington's disease (HD) is a rare genetic neurodegenerative disorder caused by the amplification of CAG repeats in the Huntington (HTT) gene and the accumulation of mutant proteins (mHTT) (Bates et al., 2015). The pathophysiology of HD remains unclear, but previous studies have highlighted the role of the immune system and neuroinflammation in HD pathology. mHTT is highly expressed in microglia and peripheral immune cells. As an inflammatory stimulus for these cells, mHTT may promote inflammatory responses through direct toxic effects, impaired glutamatergic homeostasis, or mitochondrial dysfunction (Taherzadeh-Fard et al., 2011; Weiss et al., 2012). Previous studies have demonstrated that increased microglia activation and dysregulation of astrocytic neuroinflammatory signaling pathways are associated with the progression of HD (Hsiao et al., 2013). However, peripheral adaptive immune cells rarely infiltrate into the CNS. At present, HD remains incurable, and immunotherapy and anti-inflammatory drug treatments are not effective. Many molecular targets as well as gene therapies are currently under clinical investigation (Devadiga and Bharate, 2022).

## Therapeutic Strategies for Neurodegenerative Diseases

Previous studies have demonstrated the effectiveness of strategies harnessing peripheral blood innate immune cells in clearing Aβ from brain parenchyma and blood vessels, thereby slowing the progression of AD in mouse models (Koronyo et al., 2015; Rentsendorj et al., 2018; Koronyo-Hamaoui et al., 2020). Koronyo et al. (2015) indicated that the cerebral infiltration of monocytes was beneficial to disease outcome, due to the effects on restriction of astrogliosis, cellular uptake and enzymatic degradation of Aβ. Rentsendorj et al. shown that Osteopontin (OPN), which is highly expressed in bone marrow monocytes, is an important regulator of macrophage polarization toward an anti-inflammatory immunophenotype and clearance of pathogenic Aβ (Rentsendorj et al., 2018). However there is still controversy as to whether peripheral monocytes can enter the AD brain and whether they can be used as a treatment for neurodegenerative diseases (Reed-Geaghan et al., 2020).

Adaptive immunity is also essential in the pathogenesis of neurodegenerative diseases, and the understanding of the adaptive system has a positive effect on facilitating immune-mediated treatment of neurodegenerative diseases. For example, Tregs exert neuroprotective effects by regulating microglia, effector T cells (Teffs), motor neurons (MN). Although the passive transfer of mSOD1 Tregs to ALS mice lacking functional T lymphocytes has made great progress in prolonging survival, there is still a long way to go to achieve successful clinical translation (Banerjee et al., 2008; Beers et al., 2011). A first-in-human phase 1 trial has shown that autologous Tregs infusion can slow disease progression in ALS patients. However, autologous Tregs are inefficient in targeting the CNS and require frequent and large infusions to induce therapeutic effects (Thonhoff et al., 2018).

As shown above, the pathological amyloid aggregation of α-Syn protein is the main pathological hallmark of PD, and neuroinflammation also plays an important role in the progression of PD. Modulating the activation state of glial cells by directly targeting cellular neuroinflammation, inhibiting harmful pro-inflammatory neurotoxicity, and enhancing their anti-inflammatory protective function is also a new approach to PD treatment (Subramaniam and Federoff, 2017). For example, NK cells can internalize and degrade α-syn aggregates through the endosome/lysosome pathway (Earls et al., 2020). NK cells can also interact with microglia, thereby producing cytotoxicity against hyperactive microglia (Earls and Lee, 2020). In addition, immunotherapy is also a very promising protocol for the treatment of PD, and the research on active and passive immunity against α-syn has been a novel starting point for the therapy of PD. Immunotherapy improves disease progression in patients with early-stage PD by reducing extracellular α-Syn load (Mandler et al., 2015). In clinical trials, active immunization mainly includes two humanized immunogens, PD01A and PD03A. PD passive immunization mainly includes PRX002, BIIB054 humanized antibodies, and two other anti-α-syn monoclonal antibodies MEDI1341 and BAN0805 are in the early stages of development. The active and passive immunity of α-syn has been described in detail in other literature (Zella M. A. S. et al., 2019; Zella S. M. A. et al., 2019). Recent studies demonstrated that vaccination based on DCs can provide a bridge between innate and adaptive immune responses (Brezovakova et al., 2018). By using dendritic cells as natural adjuvants, the host immune system is enhanced and the production of specific antibodies helps to clear pathological aggregation of intracellular proteins (Brezovakova et al., 2018; Sabahi et al., 2021). The vaccination based on DCs maintains the balance of the immune response and may have advantages over traditional protein-based vaccination (Sabahi et al., 2021). Since the first attempts at immunotherapy for neurodegenerative diseases, promising advances have been made, however, further studies are required to confirm its efficacy in neurodegenerative diseases.

Traditional Chinese herbal medicine (TCM) has been used for thousands of years as one of the therapies for neurodegenerative diseases, including dementia. In recent years, with the development of modern pharmacological research techniques, bioactive components isolated from TCM beneficial to patients with neurodegenerative diseases have been identified and purified, and their mechanisms of action have been extensively studied.

Previous studies have demonstrated that herbal monomers and extracts modulate AD by reducing β-amyloid production and regulating autophagy, oxidative stress, microglia polarization, and mitochondrial function (Chen S. Y. et al., 2020). For example, *Achyranthes bidentata* Blume (AB), a traditional Chinese medicine, is widely used in the treatment of dementia. ABS inhibits amyloid deposition and reduces the activation of microglia and astrocytes. It also modulates ERK and NF-κB pathways, decreases levels of proinflammatory cytokines in the brain and reduces neuroinflammation (Lin et al., 2019). Astragaloside IV (AST-IV) can exert anti-inflammatory effects on microglia by inhibiting the TLR4/NF-κB signaling pathway and promote the transformation of microglia to a neuroprotective M2 phenotype (Yu et al., 2019). In addition, QMAD, a dichloromethane soluble fraction of the Chinese herb QuMai, induces Tregs by altering intracellular signaling that restricts AKT phosphorylation (Reid-Adam et al., 2013), which may be a new strategy for the treatment of ALS.

A growing number of studies have demonstrated the crosstalk between the gut microbiota, the peripheral immune system and the CNS. Meanwhile, the dysregulation of the gut microbiota was shown to be necessary for the infiltration of peripheral immune cells into the brain. Also, studies on microglia-gut connections have suggested the important role of brain-gut microbiota in neurodegenerative diseases (Perez-Pardo et al., 2018; Wang et al., 2018). This also provides new ideas on the possible mechanisms of TCM for the treatment of neurodegenerative diseases. For example, Hua-Feng-Dan (HFD) is used to treat neurological dysfunction such as PD. The cinnabar and realgar in HFD were effective in restoring LPS and rotenone induced alterations in intestinal flora. This effect on intestinal microbes has also been shown to be associated with neuroprotective effects. HFD produces a protective effect against LPS and rotenone-induced DA neurotoxicity by delaying DA neuron loss, increasing TH protein expression, and reducing microglia activation (Chen C. et al., 2020). GV-971, prepared from marine brown algae extract, could remodel the composition of intestinal flora in AD mice, thereby inhibiting Th1 cell differentiation and M1-type microglia activation (Wang et al., 2019). These studies demonstrate that herbal monomers and compound herbs may suppress inflammatory responses in neurodegenerative diseases by regulating intestinal flora.

## Conclusion

In this review, we highlight the emerging role of the peripheral and central immune systems in neurodegenerative diseases, as well as their interactions (Table 1). In recent years, the prevalence of neurodegenerative diseases has been gradually increasing, imposing a huge economic and emotional burden on society and patients. However, there are no effective therapies to stop or reverse the progression of neurodegenerative diseases. Despite the recent progress, clinical trials so far have been disappointing. We propose in this review that for immunotherapy of neurodegenerative diseases, it is important to focus on not only CNS immunity or peripheral immunity but also their interactions. And the limitations of the current understanding of interactions between CNS and peripheral immunity are challenges that need to be urgently overcome. A detailed understanding of the key steps in the process of immune cell infiltration from the peripheral circulation to the CNS and aggressive interventions in these steps may lead to the development of more effective therapies to manage these intractable neurodegenerative diseases. In addition, the natural active ingredients in TCM have multifaceted pharmacological effects, which provide new ideas for the multi-targeted treatment of neurodegenerative diseases. It has been reported that TCM is involved in the pathogenesis of neurodegenerative diseases through multiple pathways, and future studies will be based more on the mechanism of action of TCM on the peripheral and central immune systems. A portion of TCM drugs have been applied in clinical trials, providing novel treatment strategies for neurodegenerative diseases.

**Table 1:** The role of central and peripheral immune cells on the pathogenesis of neurodegenerative diseases.

	**Amyotrophic lateral sclerosis (ALS)**	**Alzheimer's disease (AD)**	**Parkinson's disease (PD)**	**References**
Microglia	• Exerted an neuroprotective effect in the early stages of ALS. • Converted to a proinflammatory, neurotoxic state as ALS progressed.	• Activated microglia have been shown to clear excess Aβ plaques and cellular debris. • Neurotoxic and diminished phagocytosis and as AD progresses.	• Microglia contribute to the clearance of misfolded α-syn aggregates in PD. • Involved in the degeneration of dopaminergic neurons.	(Liao et al., 2012; Frakes et al., 2014; Zhao et al., 2015; Brück et al., 2016; Ries and Sastre, 2016; Hansen et al., 2018; Kim et al., 2018; Spiller et al., 2018)
Astrocytes	• Astrocytes are active participants in neuronal damage in ALS by producing neurotoxicmediators. • Regulated BBB permeability and can crosstalk with CNS immune components and infiltrating peripheral immune components.	• Reactive astrocytes are involved in AD pathology. • Astrocytes can also take up and internalize Aβ from the extracellular environment and participate in its degradation.	• Played a protective role in PD progression by isolating and degrading proinflammatory extracellular α-syn. • LRRK2- and GBA-mutant astrocytes contribute to the development of PD. • Involved in the disruption of the BBB.	(Rannikko et al., 2015; De Biase et al., 2017; Chun et al., 2020; Linnerbauer et al., 2020; Novellino et al., 2020; Sonninen et al., 2020; Lan et al., 2022)
Monocyte	• Exerted protective effects in the early stages of ALS. • Accelerated the progression of ALS during disease progression.	• Monocytes appear to have dual roles in AD pathophysiology.	• Peripheral blood mononuclear cells are dysregulated, with an increased proportion of proinflammatory monocytes. • Pathological α-syn promotes recruitment of proinflammatory monocytes to the brain.	(Naert and Rivest, 2013; Grozdanov et al., 2014; Zondler et al., 2016; Fani Maleki and Rivest, 2019; Komiya et al., 2020; Xu et al., 2022)
NK cells	• NK cells promote ALS progression in a gender- and age-specific manner.	• Infiltration of the brain by peripheral NK cells and the resulting neuroinflammatory changes have been observed in human AD and animol model.	• Internalize and degrade α-syn aggregates through the endosome/lysosome pathway. • Produce cytotoxicity against hyperactive microglia.	(Earls and Lee, 2020; Earls et al., 2020; Jin et al., 2021; Lu et al., 2021; Murdock et al., 2021)
Dendritic cells	• The role of DCs in ALS pathogenesis is still unclear.	• Dendritic cell-based immunotherapy against AD can be used as potential therapeutic approach.	• Tolerogenic bone marrow-derived DCs (BMDCs) induced Tregs.	(Brezovakova et al., 2018; Schutt et al., 2018)
T cells	• The circulating CD4+ T cells are involved in ALS progression through multiple mechanisms. In animal model, CD4+ T cells provided supportive neuroprotection.	• The role of the T cells in the development of AD remains controversial.	• Tregs exert neuroprotective effects through the interaction of the peripheral and central immune systems.	(Reynolds et al., 2007; Beers et al., 2008)
B cells	• The role of B cells in ALS pathogenesis is still unclear.	• Played an essential role on cerebral Aβ pathology. • The role of the B cells in the development of AD remains controversial.	• No B cells were identified in the post-mortem brain tissue of PD patients. • IgG deposition was found on dopaminergic neurons in the substantia nigra and Lewy bodies.	(Orr et al., 2005; Späni et al., 2015; Kim et al., 2021)

## Author Contributions

YZ contributed to the conception and design of this review and finally approved the version to be submitted. XZ, SC, JZ, and JM designed, wrote, and edited the manuscript. All authors read and revised the final manuscript.

## Conflict of Interest

The authors declare that the research was conducted in the absence of any commercial or financial relationships that could be construed as a potential conflict of interest.

## Publisher's Note

All claims expressed in this article are solely those of the authors and do not necessarily represent those of their affiliated organizations, or those of the publisher, the editors and the reviewers. Any product that may be evaluated in this article, or claim that may be made by its manufacturer, is not guaranteed or endorsed by the publisher.

## Abbreviations

AD: Alzheimer's disease
AB: *Achyranthes bidentata* Blume
ALS: Amyotrophic lateral sclerosis
APCs: antigen-presenting cells
AST-IV: Astragaloside IV
Aβ: β-amyloid
BBB: blood–brain barrier
CNS: central nervous system
CTLs: cytotoxic T lymphocytes
CX3CL1: CX3C chemokine ligand 1
DN: dopaminergic neuron
DAMPs: danger-associated molecular patterns
DCs: dendritic cells
eQTL: expressed quantitative trait loci
HD: Huntington's disease
HFD: Hua-Feng-Dan
LRRK2: Leucine-rich repeat kinase 2
MDMs: monocyte-derived macrophages
MHC-: major histocompatibility complex-
mHTT: mutant HTT
MMPs: matrix metalloproteinases
MN: motor neuron
moDCs: monocyte-derived dendritic cells
MS: multiple sclerosis
NETs: neutrophil extracellular traps
NF- κ B: nuclear factor-kappa B
NFTs: neurofibrillary tangles
NLRP3: NOD-like receptor thermal protein domain associated protein 3
NMJ: neuromuscular junction
NO: nitric oxide
NVU: neurovascular unit
OLs: oligodendrocytes
OPCs: Oligodendrocyte precursor cells
OPN: Osteopontin
PD: Parkinson's disease
ROS: reactive oxygen species
SC: spinal cord
SNpc: substantia nigra pars compact
TCM: traditional Chinese herbal medicine
Teffs: effector T cells
TGF-β1: transforming growth factor-β1
TLR: Toll-like receptors
Tregs, regulatory T: lymphocytes
VEGFA: vascular endothelial growth factor A
α-syn: alpha-synuclein.

## References

[B1] Abdel-Nour M. Carneiro L. A. M. Downey J. Tsalikis J. Outlioua A. Prescott D.. (2019). The heme-regulated inhibitor is a cytosolic sensor of protein misfolding that controls innate immune signaling. Science. 365, eaaw4144. 10.1126/science.aaw4144 31273097 PMC10433729

[B2] Agarwal D. Sandor C. Volpato V. Caffrey T. M. Monzón-Sandoval J. Bowden R.. (2020). A single-cell atlas of the human substantia nigra reveals cell-specific pathways associated with neurological disorders. Nat. Commun. 11, 4183. 10.1038/s41467-020-17876-0 32826893 PMC7442652

[B3] Ajami B. Bennett J. L. Krieger C. Tetzlaff W. Rossi F. M. (2007). Local self-renewal can sustain CNS microglia maintenance and function throughout adult life. Nat. Neurosci. 10, 1538–1543. 10.1038/nn2014 18026097

[B4] Alvarez J. I. Dodelet-Devillers A. Kebir H. Ifergan I. Fabre P. J. Terouz S.. (2011). The Hedgehog pathway promotes blood-brain barrier integrity and CNS immune quiescence. Science. 334, 1727–1731. 10.1126/science.1206936 22144466

[B5] Amor S. McNamara N. B. Gerrits E. Marzin M. C. Kooistra S. M. Miron V. E.. (2022). White matter microglia heterogeneity in the CNS. Acta Neuropathol. 143, 125–141. 10.1007/s00401-021-02389-x 34878590

[B6] Anandasabapathy N. Victora G. D. Meredith M. Feder R. Dong B. Kluger C.. (2011). Flt3L controls the development of radiosensitive dendritic cells in the meninges and choroid plexus of the steady-state mouse brain. J. Exp. Med. 208, 1695–1705. 10.1084/jem.20102657 21788405 PMC3149213

[B7] Baaklini C. S. Rawji K. S. Duncan G. J. Ho M. F. S. Plemel J. R. (2019). Central nervous system remyelination: roles of glia and innate immune cells. Front. Mol. Neurosci. 12, 225. 10.3389/fnmol.2019.00225 31616249 PMC6764409

[B8] Badimon A. Strasburger H. J. Ayata P. Chen X. Nair A. Ikegami A.. (2020). Negative feedback control of neuronal activity by microglia. Nature. 586, 417–423. 10.1038/s41586-020-2777-8 32999463 PMC7577179

[B9] Baek H. Ye M. Kang G.-H. Lee C. Lee G. Choi D. B.. (2016). Neuroprotective effects of CD4+CD25+Foxp3+ regulatory T cells in a 3xTg-AD Alzheimer's disease model. Oncotarget. 7, 69347–69357. 10.18632/oncotarget.12469 27713140 PMC5342482

[B10] Baik S. H. Cha M.-Y. Hyun Y.-M. Cho H. Hamza B. Kim D. K.. (2014). Migration of neutrophils targeting amyloid plaques in Alzheimer's disease mouse model. Neurobiol. Aging. 35, 1286–1292. 10.1016/j.neurobiolaging.2014.01.003 24485508 PMC4248665

[B11] Banerjee R. Mosley R. L. Reynolds A. D. Dhar A. Jackson-Lewis V. Gordon P. H.. (2008). Adaptive immune neuroprotection in G93A-SOD1 amyotrophic lateral sclerosis mice. PLoS ONE. 3, e2740. 10.1371/journal.pone.0002740 18648532 PMC2481277

[B12] Baruch K. Rosenzweig N. Kertser A. Deczkowska A. Sharif A. M. Spinrad A.. (2015). Breaking immune tolerance by targeting Foxp3(+) regulatory T cells mitigates Alzheimer's disease pathology. Nat. Commun. 6, 7967. 10.1038/ncomms8967 26284939 PMC4557123

[B13] Bates G. P. Dorsey R. Gusella J. F. Hayden M. R. Kay C. Leavitt B. R.. (2015). Huntington disease. Nat. Rev. Dis. Primers. 1, 15005. 10.1038/nrdp.2015.5 27188817

[B14] Becerra-Calixto A. Cardona-Gómez G. P. (2017). The role of astrocytes in neuroprotection after brain stroke: potential in cell therapy. Front. Mol. Neurosci. 10, 88. 10.3389/fnmol.2017.00088 28420961 PMC5376556

[B15] Beers D. R. Henkel J. S. Zhao W. Wang J. Appel S. H. (2008). CD4+ T cells support glial neuroprotection, slow disease progression, and modify glial morphology in an animal model of inherited ALS. Proc. Natl. Acad Sci. U S A. 105, 15558–15563. 10.1073/pnas.0807419105 18809917 PMC2547419

[B16] Beers D. R. Henkel J. S. Zhao W. Wang J. Huang A. Wen S.. (2011). Endogenous regulatory T lymphocytes ameliorate amyotrophic lateral sclerosis in mice and correlate with disease progression in patients with amyotrophic lateral sclerosis. Brain. 134, 1293–1314. 10.1093/brain/awr074 21596768 PMC3097891

[B17] Beers D. R. Zhao W. Wang J. Zhang X. Wen S. Neal D.. (2017). ALS patients' regulatory T lymphocytes are dysfunctional, and correlate with disease progression rate and severity. JCI Insight. 2, e89530. 10.1172/jci.insight.89530 28289705 PMC5333967

[B18] Benakis C. Brea D. Caballero S. Faraco G. Moore J. Murphy M.. (2016). Commensal microbiota affects ischemic stroke outcome by regulating intestinal γδ T cells. Nat. Med. 22, 516–523. 10.1038/nm.4068 27019327 PMC4860105

[B19] Boulanger J. J. Messier C. (2014). From precursors to myelinating oligodendrocytes: contribution of intrinsic and extrinsic factors to white matter plasticity in the adult brain. Neuroscience. 269, 343–366. 10.1016/j.neuroscience.2014.03.063 24721734

[B20] Braak H. Sastre M. Del Tredici K. (2007). Development of alpha-synuclein immunoreactive astrocytes in the forebrain parallels stages of intraneuronal pathology in sporadic Parkinson's disease. Acta Neuropathol. 114, 231–241. 10.1007/s00401-007-0244-3 17576580

[B21] Brezovakova V. Valachova B. Hanes J. Novak M. Jadhav S. (2018). Dendritic cells as an alternate approach for treatment of neurodegenerative disorders. Cell Mol. Neurobiol. 38, 1207–1214. 10.1007/s10571-018-0598-1 29948552 PMC11481983

[B22] Brochard V. Combadière B. Prigent A. Laouar Y. Perrin A. Beray-Berthat V.. (2009). Infiltration of CD4+ lymphocytes into the brain contributes to neurodegeneration in a mouse model of Parkinson disease. J. Clin. Invest. 119, 182–192. 10.1172/JCI36470 19104149 PMC2613467

[B23] Brown R. H. Al-Chalabi A. (2017). Amyotrophic lateral sclerosis. N. Engl. J. Med. 377, 162–172. 10.1056/NEJMra1603471 28700839

[B24] Brück D. Wenning G. K. Stefanova N. Fellner L. (2016). Glia and alpha-synuclein in neurodegeneration: A complex interaction. Neurobiol. Dis. 85, 262–274. 10.1016/j.nbd.2015.03.003 25766679 PMC4730552

[B25] Bryois J. Skene N. G. Hansen T. F. Kogelman L. J. A. Watson H. J. Liu Z.. (2020). Genetic identification of cell types underlying brain complex traits yields insights into the etiology of Parkinson's disease. Nat. Genet. 52, 482–493. 10.1038/s41588-020-0610-9 32341526 PMC7930801

[B26] Bulloch K. Miller M. M. Gal-Toth J. Milner T. A. Gottfried-Blackmore A. Waters E. M.. (2008). CD11c/EYFP transgene illuminates a discrete network of dendritic cells within the embryonic, neonatal, adult, and injured mouse brain. J. Comp. Neurol. 508, 687–710. 10.1002/cne.21668 18386786

[B27] Burgaletto C. Munaf,ò A. Di Benedetto G. De Francisci C. Caraci F. Di Mauro R.. (2020). The immune system on the TRAIL of Alzheimer's disease. J. Neuroinflammation. 17, 298. 10.1186/s12974-020-01968-1 33050925 PMC7556967

[B28] Cai B. Seong K.-J. Bae S.-W. Chun C. Kim W.-J. Jung J.-Y. (2018). A synthetic diosgenin primary amine derivative attenuates LPS-stimulated inflammation via inhibition of NF-κB and JNK MAPK signaling in microglial BV2 cells. Int. Immunopharmacol. 61, 204–214. 10.1016/j.intimp.2018.05.021 29890414

[B29] Cao S. Fisher D. W. Rodriguez G. Yu T. Dong H. (2021). Comparisons of neuroinflammation, microglial activation, and degeneration of the locus coeruleus-norepinephrine system in APP/PS1 and aging mice. J. Neuroinflamm. 18, 10. 10.1186/s12974-020-02054-2 33407625 PMC7789762

[B30] Castellani G. Schwartz M. (2020). Immunological features of non-neuronal brain cells: implications for Alzheimer's disease immunotherapy. Trends Immunol. 41, 794–804. 10.1016/j.it.2020.07.005 32800704

[B31] Chen C. Zhang B.-B. Hu A.-L. Li H. Liu J. Zhang F. (2020). Protective role of cinnabar and realgar in Hua-Feng-Dan against LPS plus rotenone-induced neurotoxicity and disturbance of gut microbiota in rats. J. Ethnopharmacol. 247, 112299. 10.1016/j.jep.2019.112299 31606537

[B32] Chen S. Y. Gao Y. Sun J. Y. Meng X. L. Yang D. Fan L. H.. (2020). Traditional chinese medicine: role in reducing beta-amyloid, apoptosis, autophagy, neuroinflammation, oxidative stress, and mitochondrial dysfunction of Alzheimer's disease. Front. Pharmacol. 11, 497. 10.3389/fphar.2020.00497 32390843 PMC7188934

[B33] Chen X. Feng W. Huang R. Guo X. Chen Y. Zheng Z.. (2014). Evidence for peripheral immune activation in amyotrophic lateral sclerosis. J. Neurol. Sci. 347, 90–95. 10.1016/j.jns.2014.09.025 25312013

[B34] Chen X.-Q. Mobley W. C. (2019). Alzheimer disease pathogenesis: insights from molecular and cellular biology studies of oligomeric Aβ and tau species. Front. Neurosci. 13, 659. 10.3389/fnins.2019.00659 31293377 PMC6598402

[B35] Chu T. Shields L. B. E. Zeng W. Zhang Y. P. Wang Y. Barnes G. N.. (2021). Dynamic glial response and crosstalk in demyelination-remyelination and neurodegeneration processes. Neural. Regen. Res. 16, 1359–1368. 10.4103/1673-5374.300975 33318418 PMC8284258

[B36] Chun H. Im H. Kang Y. J. Kim Y. Shin J. H. Won W.. (2020). Severe reactive astrocytes precipitate pathological hallmarks of Alzheimer's disease via HO production. Nat. Neurosci. 23, 1555–1566. 10.1038/s41593-020-00735-y 33199896

[B37] Ciccocioppo F. Bologna G. Ercolino E. Pierdomenico L. Simeone P. Lanuti P.. (2020). Neurodegenerative diseases as proteinopathies-driven immune disorders. Neural Regen. Res. 15, 850–856. 10.4103/1673-5374.268971 31719246 PMC6990794

[B38] Colton C. A. (2013). Immune heterogeneity in neuroinflammation: dendritic cells in the brain. J. Neuroimmune. Pharmacol. 8, 145–162. 10.1007/s11481-012-9414-8 23114889 PMC4279719

[B39] Coque E. Salsac C. Espinosa-Carrasco G. Varga B. Degauque N. Cadoux M.. (2019). Cytotoxic CD8 T lymphocytes expressing ALS-causing SOD1 mutant selectively trigger death of spinal motoneurons. Proc. Natl. Acad Sci. U S A. 116, 2312–2317. 10.1073/pnas.1815961116 30674678 PMC6369778

[B40] Croese T. Castellani G. Schwartz M. (2021). Immune cell compartmentalization for brain surveillance and protection. Nat. Immunol. 22, 1083–1092. 10.1038/s41590-021-00994-2 34429552

[B41] Cserép C. Pósfai B. Dénes Á. (2021). Shaping neuronal fate: functional heterogeneity of direct microglia-neuron interactions. Neuron. 109, 222–240. 10.1016/j.neuron.2020.11.007 33271068

[B42] Cserép C. Pósfai B. Lénárt N. Fekete R. Lászl,ó Z. I. Lele Z.. (2020). Microglia monitor and protect neuronal function through specialized somatic purinergic junctions. Science. 367, 528–537. 10.1126/science.aax6752 31831638

[B43] Dai L. Shen Y. (2021). Insights into T-cell dysfunction in Alzheimer's disease. Aging Cell. 20, e13511. 10.1111/acel.13511 34725916 PMC8672785

[B44] D'Ambrosi N. Apolloni S. (2020). Fibrotic scar in neurodegenerative diseases. Front. Immunol. 11, 1394. 10.3389/fimmu.2020.01394 32922384 PMC7456854

[B45] Dansokho C. Ait Ahmed D. Aid S. Toly-Ndour C. Chaigneau T. Calle V.. (2016). Regulatory T cells delay disease progression in Alzheimer-like pathology. Brain. 139, 1237–1251. 10.1093/brain/awv408 26912648

[B46] Dardalhon V. Korn T. Kuchroo V. K. Anderson A. C. (2008). Role of Th1 and Th17 cells in organ-specific autoimmunity. J. Autoimmun. 31, 252–256. 10.1016/j.jaut.2008.04.017 18502610 PMC3178062

[B47] Davey M. S. Willcox C. R. Hunter S. Kasatskaya S. A. Remmerswaal E. B. M. Salim M.. (2018). The human Vδ2 T-cell compartment comprises distinct innate-like Vγ9 and adaptive Vγ9 subsets. Nat. Commun. 9, 1760. 10.1038/s41467-018-04076-0 29720665 PMC5932074

[B48] Davey M. S. Willcox C. R. Joyce S. P. Ladell K. Kasatskaya S. A. McLaren J. E.. (2017). Clonal selection in the human Vδ1 T cell repertoire indicates γδ TCR-dependent adaptive immune surveillance. Nat. Commun. 8, 14760. 10.1038/ncomms14760 28248310 PMC5337994

[B49] De Biase L. M. Schuebel K. E. Fusfeld Z. H. Jair K. Hawes I. A. Cimbro R.. (2017). Local Cues Establish and Maintain Region-Specific Phenotypes of Basal Ganglia Microglia. Neuron. 95, 341*356.e346. 10.1016/j.neuron.2017.06.020 28689984 PMC5754189

[B50] de Boer E. M. J. Orie V. K. Williams T. Baker M. R. De Oliveira H. M. Polvikoski T.. (2021). TDP-43 proteinopathies: a new wave of neurodegenerative diseases. J. Neurol. Neurosurg. Psychiat. 92, 86–95. 10.1136/jnnp-2020-322983 33177049 PMC7803890

[B51] Deniston C. K. Salogiannis J. Mathea S. Snead D. M. Lahiri I. Matyszewski M.. (2020). Structure of LRRK2 in Parkinson's disease and model for microtubule interaction. Nature. 588, 344–349. 10.1038/s41586-020-2673-2 32814344 PMC7726071

[B52] Devadiga S. J. Bharate S. S. (2022). Recent developments in the management of Huntington's disease. Bioorg. Chem. 120, 105642. 10.1016/j.bioorg.2022.105642 35121553

[B53] Ding X. Gu R. Zhang M. Ren H. Shu Q. Xu G.. (2018). Microglia enhanced the angiogenesis, migration and proliferation of co-cultured RMECs. BMC Ophthalmol. 18, 249. 10.1186/s12886-018-0886-z 30223824 PMC6142340

[B54] Dong Y. Lagarde J. Xicota L. Corne H. Chantran Y. Chaigneau T.. (2018). Neutrophil hyperactivation correlates with Alzheimer's disease progression. Ann. Neurol. 83, 387–405. 10.1002/ana.25159 29369398

[B55] Earls R. H. Lee J. K. (2020). The role of natural killer cells in Parkinson's disease. Exp. Mol. Med. 52, 1517–1525. 10.1038/s12276-020-00505-7 32973221 PMC8080760

[B56] Earls R. H. Menees K. B. Chung J. Gutekunst C.-A. Lee H. J. Hazim M. G.. (2020). NK cells clear α-synuclein and the depletion of NK cells exacerbates synuclein pathology in a mouse model of α-synucleinopathy. Proc. Natl. Acad Sci. U S A. 117, 1762–1771. 10.1073/pnas.1909110117 31900358 PMC6983411

[B57] Elamin M. Bede P. Byrne S. Jordan N. Gallagher L. Wynne B.. (2013). Cognitive changes predict functional decline in ALS: a population-based longitudinal study. Neurology. 80, 1590–1597. 10.1212/WNL.0b013e31828f18ac 23553481

[B58] Ellwardt E. Walsh J. T. Kipnis J. Zipp F. (2016). Understanding the role of T Cells in CNS homeostasis. Trends Immunol. 37, 154–165. 10.1016/j.it.2015.12.008 26775912

[B59] Endo F. Komine O. Fujimori-Tonou N. Katsuno M. Jin S. Watanabe S.. (2015). Astrocyte-derived TGF-β1 accelerates disease progression in ALS mice by interfering with the neuroprotective functions of microglia and T cells. Cell Rep. 11, 592–604. 10.1016/j.celrep.2015.03.053 25892237

[B60] Engelhardt B. Vajkoczy P. Weller R. O. (2017). The movers and shapers in immune privilege of the CNS. Nat. Immunol. 18, 123–131. 10.1038/ni.3666 28092374

[B61] Faissner S. Plemel J. R. Gold R. Yong V. W. (2019). Progressive multiple sclerosis: from pathophysiology to therapeutic strategies. Nat. Rev. Drug Discov. 18, 905–922. 10.1038/s41573-019-0035-2 31399729

[B62] Falcão A. M. van Bruggen D. Marques S. Meijer M. Jäkel S. Agirre E.. (2018). Disease-specific oligodendrocyte lineage cells arise in multiple sclerosis. Nat. Med. 24, 1837–1844. 10.1038/s41591-018-0236-y 30420755 PMC6544508

[B63] Fani Maleki A. Rivest S. (2019). Innate Immune Cells: Monocytes, Monocyte-Derived Macrophages and Microglia as Therapeutic Targets for Alzheimer's Disease and Multiple Sclerosis. Front. Cell Neurosci. 13, 355. 10.3389/fncel.2019.00355 31427930 PMC6690269

[B64] Frakes A. E. Ferraiuolo L. Haidet-Phillips A. M. Schmelzer L. Braun L. Miranda C. J.. (2014). Microglia induce motor neuron death via the classical NF-κB pathway in amyotrophic lateral sclerosis. Neuron. 81, 1009–1023. 10.1016/j.neuron.2014.01.013 24607225 PMC3978641

[B65] Galiano-Landeira J. Torra A. Vila M. Bov,é J. (2020). CD8 T cell nigral infiltration precedes synucleinopathy in early stages of Parkinson's disease. Brain. 143, 3717–3733. 10.1093/brain/awaa269 33118032

[B66] Garofalo S. Cocozza G. Porzia A. Inghilleri M. Raspa M. Scavizzi F.. (2020). Natural killer cells modulate motor neuron-immune cell cross talk in models of Amyotrophic Lateral Sclerosis. Nat. Commun. 11, 1773. 10.1038/s41467-020-15644-8 32286313 PMC7156729

[B67] Gate D. Saligrama N. Leventhal O. Yang A. C. Unger M. S. Middeldorp J.. (2020). Clonally expanded CD8 T cells patrol the cerebrospinal fluid in Alzheimer's disease. Nature. 577, 399–404. 10.1038/s41586-019-1895-7 31915375 PMC7445078

[B68] Gendelman H. E. Appel S. H. (2011). Neuroprotective activities of regulatory T cells. Trends Mol. Med. 17, 687–688. 10.1016/j.molmed.2011.08.005 21996344 PMC5892451

[B69] Giannoni P. Claeysen S. Noe F. Marchi N. (2020). Peripheral routes to neurodegeneration: passing through the blood-brain barrier. Front. Aging Neurosci. 12, 3. 10.3389/fnagi.2020.00003 32116645 PMC7010934

[B70] Gomez Perdiguero E. Klapproth K. Schulz C. Busch K. Azzoni E. Crozet L.. (2015). Tissue-resident macrophages originate from yolk-sac-derived erythro-myeloid progenitors. Nature. 518, 547–551. 10.1038/nature13989 25470051 PMC5997177

[B71] Greenhalgh A. D. David S. Bennett F. C. (2020). Immune cell regulation of glia during CNS injury and disease. Nat. Rev. Neurosci. 21, 139–152. 10.1038/s41583-020-0263-9 32042145

[B72] Greenhalgh A. D. Zarruk J. G. Healy L. M. Baskar Jesudasan S. J. Jhelum P. Salmon C. K.. (2018). Peripherally derived macrophages modulate microglial function to reduce inflammation after CNS injury. PLoS Biol. 16, e2005264. 10.1371/journal.pbio.2005264 30332405 PMC6205650

[B73] Griffin D. E. Metcalf T. (2011). Clearance of virus infection from the CNS. Curr. Opin. Virol. 1, 216–221. 10.1016/j.coviro.2011.05.021 21927638 PMC3171972

[B74] Groh J. Knöpper K. Arampatzi P. Yuan X. Lößlein L. Saliba A.-E.. (2021). Accumulation of cytotoxic T cells in the aged CNS leads to axon degeneration and contributes to cognitive and motor decline. Nat. Aging. 1, 357–367. 10.1038/s43587-021-00049-z 37117598

[B75] Grozdanov V. Bliederhaeuser C. Ruf W. P. Roth V. Fundel-Clemens K. Zondler L.. (2014). Inflammatory dysregulation of blood monocytes in Parkinson's disease patients. Acta Neuropathol. 128, 651–663. 10.1007/s00401-014-1345-4 25284487 PMC4201759

[B76] Guillerey C. Huntington N. D. Smyth M. J. (2016). Targeting natural killer cells in cancer immunotherapy. Nat. Immunol. 17, 1025–1036. 10.1038/ni.3518 27540992

[B77] Guo M. Wang J. Zhao Y. Feng Y. Han S. Dong Q.. (2020). Microglial exosomes facilitate α-synuclein transmission in Parkinson's disease. Brain. 143, 1476–1497. 10.1093/brain/awaa090 32355963 PMC7241957

[B78] Guo Y. Zeng H. Gao C. (2021). The role of neutrophil extracellular traps in central nervous system diseases and prospects for clinical application. Oxid. Med. Cell Longev. 2021, 9931742. 10.1155/2021/9931742 34336122 PMC8294981

[B79] Gustot A. Gallea J. I. Sarroukh R. Celej M. S. Ruysschaert J.-M. Raussens V. (2015). Amyloid fibrils are the molecular trigger of inflammation in Parkinson's disease. Biochem. J. 471, 323–333. 10.1042/BJ20150617 26272943

[B80] Hamann I. Unterwalder N. Cardona A. E. Meisel C. Zipp F. Ransohoff R. M.. (2011). Analyses of phenotypic and functional characteristics of CX3CR1-expressing natural killer cells. Immunology. 133, 62–73. 10.1111/j.1365-2567.2011.03409.x 21320123 PMC3088968

[B81] Hansen D. V. Hanson J. E. Sheng M. (2018). Microglia in Alzheimer's disease. J. Cell Biol. 217, 459–472. 10.1083/jcb.201709069 29196460 PMC5800817

[B82] Hardiman O. Al-Chalabi A. Chio A. Corr E. M. Logroscino G. Robberecht W.. (2017). Amyotrophic lateral sclerosis. Nat. Rev. Dis. Primers. 3, 17071. 10.1038/nrdp.2017.71 28980624

[B83] Hartl F. U. (2017). Protein Misfolding Diseases. Annu. Rev. Biochem. 86, 21–26. 10.1146/annurev-biochem-061516-044518 28441058

[B84] Haseeb M. Javaid N. Yasmeen F. Jeong U. Han J. H. Yoon J.. (2022). Novel small-molecule inhibitor of NLRP3 inflammasome reverses cognitive impairment in an Alzheimer's disease model. ACS Chem. Neurosci. 13, 818–833. 10.1021/acschemneuro.1c00831 35196855

[B85] Hazenberg M. D. Spits H. (2014). Human innate lymphoid cells. Blood. 124, 700–709. 10.1182/blood-2013-11-427781 24778151

[B86] Healy L. M. Stratton J. A. Kuhlmann T. Antel J. (2022). The role of glial cells in multiple sclerosis disease progression. Nat Rev Neurol. 10.1038/s41582-022-00624-x 35190704

[B87] Henkel J. S. Engelhardt J. I. Siklos L. Simpson E. P. Kim S. H. Pan T.. (2004). Presence of dendritic cells, MCP-1, and activated microglia/macrophages in amyotrophic lateral sclerosis spinal cord tissue. Ann. Neurol. 55, 221–235. 10.1002/ana.10805 14755726

[B88] Hertwig L. Hamann I. Romero-Suarez S. Millward J. M. Pietrek R. Chanvillard C.. (2016). CX3CR1-dependent recruitment of mature NK cells into the central nervous system contributes to control autoimmune neuroinflammation. Eur. J. Immunol. 46, 1984–1996. 10.1002/eji.201546194 27325505 PMC5020316

[B89] Hickman S. E. Allison E. K. El Khoury J. (2008). Microglial dysfunction and defective beta-amyloid clearance pathways in aging Alzheimer's disease mice. J. Neurosci. 28, 8354–8360. 10.1523/JNEUROSCI.0616-08.2008 18701698 PMC2597474

[B90] Hill A. E. Wade-Martins R. Burnet P. W. J. (2021). What is our understanding of the influence of gut microbiota on the pathophysiology of Parkinson's disease? Front. Neurosci. 15, 708587. 10.3389/fnins.2021.708587 34512244 PMC8432298

[B91] Hsiao H.-Y. Chen Y.-C. Chen H.-M. Tu P.-H. Chern Y. (2013). A critical role of astrocyte-mediated nuclear factor-κB-dependent inflammation in Huntington's disease. Hum. Mol. Genet. 22, 1826–1842. 10.1093/hmg/ddt036 23372043

[B92] Huang D. Shi F. D. Jung S. Pien G. C. Wang J. Salazar-Mather T. P.. (2006). The neuronal chemokine CX3CL1/fractalkine selectively recruits NK cells that modify experimental autoimmune encephalomyelitis within the central nervous system. FASEB J. 20, 896–905. 10.1096/fj.05-5465com 16675847

[B93] Ishii M. Iadecola C. (2020). Risk factor for Alzheimer's disease breaks the blood-brain barrier. Nature. 581, 31–32. 10.1038/d41586-020-01152-8 32350425 PMC8018585

[B94] Jeon M.-T. Kim K.-S. Kim E. S. Lee S. Kim J. Hoe H.-S.. (2021). Emerging pathogenic role of peripheral blood factors following BBB disruption in neurodegenerative disease. Ageing Res. Rev. 68, 101333. 10.1016/j.arr.2021.101333 33774194

[B95] Jin M. Günther R. Akgün K. Hermann A. Ziemssen T. (2020). Peripheral proinflammatory Th1/Th17 immune cell shift is linked to disease severity in amyotrophic lateral sclerosis. Sci. Rep. 10, 5941. 10.1038/s41598-020-62756-8 32246039 PMC7125229

[B96] Jin W.-N. Shi K. He W. Sun J.-H. Van Kaer L. Shi F.-D.. (2021). Neuroblast senescence in the aged brain augments natural killer cell cytotoxicity leading to impaired neurogenesis and cognition. Nat. Neurosci. 24, 61–73. 10.1038/s41593-020-00745-w 33257875

[B97] Kebir H. Kreymborg K. Ifergan I. Dodelet-Devillers A. Cayrol R. Bernard M.. (2007). Human TH17 lymphocytes promote blood-brain barrier disruption and central nervous system inflammation. Nat. Med. 13, 1173–1175. 10.1038/nm1651 17828272 PMC5114125

[B98] Kim K. Wang X. Ragonnaud E. Bodogai M. Illouz T. DeLuca M.. (2021). Therapeutic B-cell depletion reverses progression of Alzheimer's disease. Nat. Commun. 12, 2185. 10.1038/s41467-021-22479-4 33846335 PMC8042032

[B99] Kim K. S. Marcogliese P. C. Yang J. Callaghan S. M. Resende V. Abdel-Messih E.. (2018). Regulation of myeloid cell phagocytosis by LRRK2 via WAVE2 complex stabilization is altered in Parkinson's disease. Proc. Natl. Acad Sci. U S A. 115, E5164–E5173. 10.1073/pnas.1718946115 29760073 PMC5984500

[B100] Kobo H. Bar-Shira A. Dahary D. Gan-Or Z. Mirelman A. Goldstein O.. (2016). Down-regulation of B cell-related genes in peripheral blood leukocytes of Parkinson's disease patients with and without GBA mutations. Mol. Genet. Metab. 117, 179–185. 10.1016/j.ymgme.2015.09.005 26410072

[B101] Kolaczkowska E. Kubes P. (2013). Neutrophil recruitment and function in health and inflammation. Nat. Rev. Immunol. 13, 159–175. 10.1038/nri3399 23435331

[B102] Komiya H. Takeuchi H. Ogawa Y. Hatooka Y. Takahashi K. Katsumoto A.. (2020). CCR2 is localized in microglia and neurons, as well as infiltrating monocytes, in the lumbar spinal cord of ALS mice. Mol. Brain. 13, 64. 10.1186/s13041-020-00607-3 32349774 PMC7191738

[B103] Koronyo Y. Salumbides B. C. Sheyn J. Pelissier L. Li S. Ljubimov V.. (2015). Therapeutic effects of glatiramer acetate and grafted CD115(+) monocytes in a mouse model of Alzheimer's disease. Brain. 138, 2399–2422. 10.1093/brain/awv150 26049087 PMC4840949

[B104] Koronyo-Hamaoui M. Sheyn J. Hayden E. Y. Li S. Fuchs D.-T. Regis G. C.. (2020). Peripherally derived angiotensin converting enzyme-enhanced macrophages alleviate Alzheimer-related disease. Brain. 143, 336–358. 10.1093/brain/awz364 31794021 PMC6935752

[B105] Lan G. Wang P. Chan R. B. Liu Z. Yu Z. Liu X.. (2022). Astrocytic VEGFA: An essential mediator in blood-brain-barrier disruption in Parkinson's disease. Glia. 70, 337–353. 10.1002/glia.24109 34713920

[B106] Lassmann H. (2018). Pathogenic mechanisms associated with different clinical courses of multiple sclerosis. Front. Immunol. 9, 3116. 10.3389/fimmu.2018.03116 30687321 PMC6335289

[B107] Laurent C. Dorothée G. Hunot S. Martin E. Monnet Y. Duchamp M.. (2017). Hippocampal T cell infiltration promotes neuroinflammation and cognitive decline in a mouse model of tauopathy. Brain. 140, 184–200. 10.1093/brain/aww270 27818384 PMC5382942

[B108] Le Page A. Bourgade K. Lamoureux J. Frost E. Pawelec G. Larbi A.. (2015). NK cells are activated in amnestic mild cognitive impairment but not in mild Alzheimer's disease patients. J. Alzheimers Dis. 46, 93–107. 10.3233/JAD-143054 25720398

[B109] Le Page A. Dupuis G. Frost E. H. Larbi A. Pawelec G. Witkowski J. M.. (2018). Role of the peripheral innate immune system in the development of Alzheimer's disease. Exp. Gerontol. 107, 59–66. 10.1016/j.exger.2017.12.019 29275160

[B110] Li C. Chen Y.-H. Zhang K. (2020). Neuroprotective Properties and Therapeutic Potential of Bone Marrow-Derived Microglia in Alzheimer's Disease. Am. J. Alzheimers Dis. Other Demen. 35, 1533317520927169. 10.1177/1533317520927169 32536247 PMC10623913

[B111] Li Y. Xia Y. Yin S. Wan F. Hu J. Kou L.. (2021). Targeting microglial α-Synuclein/TLRs/NF-kappaB/NLRP3 inflammasome axis in Parkinson's disease. Front. Immunol. 12, 719807. 10.3389/fimmu.2021.719807 34691027 PMC8531525

[B112] Liao B. Zhao W. Beers D. R. Henkel J. S. Appel S. H. (2012). Transformation from a neuroprotective to a neurotoxic microglial phenotype in a mouse model of ALS. Exp. Neurol. 237, 147–152. 10.1016/j.expneurol.2012.06.011 22735487 PMC4126417

[B113] Liddelow S. A. Guttenplan K. A. Clarke L. E. Bennett F. C. Bohlen C. J. Schirmer L.. (2017). Neurotoxic reactive astrocytes are induced by activated microglia. Nature. 541, 481–487. 10.1038/nature21029 28099414 PMC5404890

[B114] Lin L.-W. Tsai F.-H. Lan W.-C. Cheng Y.-D. Lee S.-C. Wu C.-R. (2019). Steroid-enriched fraction of achyranthes bidentata protects amyloid β peptide 1-40-induced cognitive dysfunction and neuroinflammation in rats. Mol. Neurobiol. 56, 5671–5688. 10.1007/s12035-018-1436-7 30666561

[B115] Linnerbauer M. Wheeler M. A. Quintana F. J. (2020). Astrocyte Crosstalk in CNS Inflammation. Neuron. 108, 608–622. 10.1016/j.neuron.2020.08.012 32898475 PMC7704785

[B116] Liu Z. Cheng X. Zhong S. Zhang X. Liu C. Liu F.. (2020). Peripheral and central nervous system immune response crosstalk in amyotrophic lateral sclerosis. Front. Neurosci. 14, 575. 10.3389/fnins.2020.00575 32612503 PMC7308438

[B117] Lu Y. Li K. Hu Y. Wang X. (2021). Expression of immune related genes and possible regulatory mechanisms in Alzheimer's disease. Front. Immunol. 12, 768966. 10.3389/fimmu.2021.768966 34804058 PMC8602845

[B118] Machado-Santos J. Saji E. Tröscher A. R. Paunovic M. Liblau R. Gabriely G.. (2018). The compartmentalized inflammatory response in the multiple sclerosis brain is composed of tissue-resident CD8+ T lymphocytes and B cells. Brain. 141, 2066–2082. 10.1093/brain/awy151 29873694 PMC6022681

[B119] Manda-Handzlik A. Demkow U. (2019). The brain entangled: the contribution of neutrophil extracellular traps to the diseases of the central nervous system. Cells. 8, 1477. 10.3390/cells8121477 31766346 PMC6953104

[B120] Mandler M. Valera E. Rockenstein E. Mante M. Weninger H. Patrick C.. (2015). Active immunization against alpha-synuclein ameliorates the degenerative pathology and prevents demyelination in a model of multiple system atrophy. Mol. Neurodeg. 10, 10. 10.1186/s13024-015-0008-9 25886309 PMC4411775

[B121] Marogianni C. Sokratous M. Dardiotis E. Hadjigeorgiou G. M. Bogdanos D. Xiromerisiou G. (2020). Neurodegeneration and inflammation-an interesting interplay in Parkinson's disease. Int. J. Mol. Sci. 21, 8421. 10.3390/ijms21228421 33182554 PMC7697354

[B122] Marsh S. E. Abud E. M. Lakatos A. Karimzadeh A. Yeung S. T. Davtyan H.. (2016). The adaptive immune system restrains Alzheimer's disease pathogenesis by modulating microglial function. Proc. Natl. Acad Sci. U S A. 113, E1316–E1325. 10.1073/pnas.1525466113 26884167 PMC4780638

[B123] Mayne K. White J. A. McMurran C. E. Rivera F. J. de la Fuente A. G. (2020). Aging and neurodegenerative disease: is the adaptive immune system a friend or foe? Front. Aging Neurosci. 12, 572090. 10.3389/fnagi.2020.572090 33173502 PMC7538701

[B124] McAlpine C. S. Park J. Griciuc A. Kim E. Choi S. H. Iwamoto Y.. (2021). Astrocytic interleukin-3 programs microglia and limits Alzheimer's disease. Nature. 595, 701–706. 10.1038/s41586-021-03734-6 34262178 PMC8934148

[B125] McGeer P. L. Itagaki S. Boyes B. E. McGeer E. G. (1988). Reactive microglia are positive for HLA-DR in the substantia nigra of Parkinson's and Alzheimer's disease brains. Neurology. 38, 1285–1291. 10.1212/wnl.38.8.1285 3399080

[B126] McMenamin P. G. Wealthall R. J. Deverall M. Cooper S. J. Griffin B. (2003). Macrophages and dendritic cells in the rat meninges and choroid plexus: three-dimensional localisation by environmental scanning electron microscopy and confocal microscopy. Cell Tissue. Res. 313, 259–269. 10.1007/s00441-003-0779-0 12920643

[B127] Mészáros Á. Molnár K. Nógrádi B. Hernádi Z. Nyúl-Tóth Á. Wilhelm I.. (2020). Neurovascular inflammaging in health and disease. Cells. 9, 1614. 10.3390/cells9071614 32635451 PMC7407516

[B128] Monsonego A. Zota V. Karni A. Krieger J. I. Bar-Or A. Bitan G.. (2003). Increased T cell reactivity to amyloid beta protein in older humans and patients with Alzheimer disease. J. Clin. Invest. 112, 415–422. 10.1172/JCI200318104 12897209 PMC166296

[B129] Moodley K. Angel C. E. Glass M. Graham E. S. (2011). Real-time profiling of NK cell killing of human astrocytes using xCELLigence technology. J. Neurosci. Methods. 200, 173–180. 10.1016/j.jneumeth.2011.07.005 21781988

[B130] Morgan J. Muskat K. Tippalagama R. Sette A. Burel J. Lindestam Arlehamn C. S. (2021). Classical CD4 T cells as the cornerstone of antimycobacterial immunity. Immunol. Rev. 301, 10–29. 10.1111/imr.12963 33751597 PMC8252593

[B131] Mrdjen D. Pavlovic A. Hartmann F. J. Schreiner B. Utz S. G. Leung B. P.. (2018). High-dimensional single-cell mapping of central nervous system immune cells reveals distinct myeloid subsets in health, aging, and disease. Immunity. 48, 380–395.e386. 10.1016/j.immuni.2018.01.011 29426702

[B132] Muffat J. Li Y. Yuan B. Mitalipova M. Omer A. Corcoran S.. (2016). Efficient derivation of microglia-like cells from human pluripotent stem cells. Nat. Med. 22, 1358–1367. 10.1038/nm.4189 27668937 PMC5101156

[B133] Murdock B. J. Famie J. P. Piecuch C. E. Raue K. D. Mendelson F. E. Pieroni C. H.. (2021). NK cells associate with ALS in a sex- and age-dependent manner. JCI Insight. 6, e147129. 33974561 10.1172/jci.insight.147129 PMC8262328

[B134] Naegele M. Tillack K. Reinhardt S. Schippling S. Martin R. Sospedra M. (2012). Neutrophils in multiple sclerosis are characterized by a primed phenotype. J. Neuroimmunol. 242, 60–71. 10.1016/j.jneuroim.2011.11.009 22169406

[B135] Naert G. Rivest S. (2013). A deficiency in CCR2+ monocytes: the hidden side of Alzheimer's disease. J. Mol. Cell Biol. 5, 284–293. 10.1093/jmcb/mjt028 23892208

[B136] Nardo G. Trolese M. C. de Vito G. Cecchi R. Riva N. Dina G.. (2016). Immune response in peripheral axons delays disease progression in SOD1(G93A) mice. J. Neuroinflammation. 13, 261. 10.1186/s12974-016-0732-2 27717377 PMC5055725

[B137] Nardo G. Trolese M. C. Verderio M. Mariani A. de Paola M. Riva N.. (2018). Counteracting roles of MHCI and CD8(+) T cells in the peripheral and central nervous system of ALS SOD1(G93A) mice. Mol. Neurodegener. 13, 42. 10.1186/s13024-018-0271-7 30092791 PMC6085701

[B138] Neely S.A. Williamson J.M. Klingseisen A. Zoupi L. Early J.J. Williams A.. (2022). New oligodendrocytes exhibit more abundant and accurate myelin regeneration than those that survive demyelination. Nat Neurosci. Online ahead of print. 10.1038/s41593-021-01009-x 35165460 PMC7612594

[B139] Nikolakopoulou A. M. Montagne A. Kisler K. Dai Z. Wang Y. Huuskonen M. T.. (2019). Pericyte loss leads to circulatory failure and pleiotrophin depletion causing neuron loss. Nat. Neurosci. 22, 1089–1098. 10.1038/s41593-019-0434-z 31235908 PMC6668719

[B140] Nimmerjahn A. Kirchhoff F. Helmchen F. (2005). Resting microglial cells are highly dynamic surveillants of brain parenchyma in vivo. Science. 308, 1314–1318. 10.1126/science.1110647 15831717

[B141] Nortley R. Korte N. Izquierdo P. Hirunpattarasilp C. Mishra A. Jaunmuktane Z.. (2019). Amyloid β oligomers constrict human capillaries in Alzheimer's disease via signaling to pericytes. Science. 365, eaav9518. 10.1126/science.aav9518 31221773 PMC6658218

[B142] Novellino F. Sacca V. Donato A. Zaffino P. Spadea M. F. Vismara M.. (2020). Innate immunity: a common denominator between neurodegenerative and neuropsychiatric diseases. Int. J. Mol. Sci. 21, 1115. 10.3390/ijms21031115 32046139 PMC7036760

[B143] Nyúl-Tóth Á. Kozma M. Nagyoszi P. Nagy K. Fazakas C. Hask,ó J.. (2017). Expression of pattern recognition receptors and activation of the non-canonical inflammasome pathway in brain pericytes. Brain Behav. Immun. 64, 220–231. 10.1016/j.bbi.2017.04.010 28432035

[B144] Orr C. F. Rowe D. B. Mizuno Y. Mori H. Halliday G. M. (2005). A possible role for humoral immunity in the pathogenesis of Parkinson's disease. Brain. 128, 2665–2674. 10.1093/brain/awh625 16219675

[B145] Ouali Alami N. Schurr C. Olde Heuvel F. Tang L. Y. Li Q. Tasdogan A.. (2018). NF-kappa B activation in astrocytes drives a stage-specific beneficial neuroimmunological response in ALS. Embo. J. 37, e98697. 10.15252/embj.201798697 29875132 PMC6092622

[B146] Perez-Pardo P. Dodiya H. B. Engen P. A. Naqib A. Forsyth C. B. Green S. J.. (2018). Gut bacterial composition in a mouse model of Parkinson's disease. Benef. Microbes. 9, 799–814. 10.3920/BM2017.0202 30099890

[B147] Ponomarev E. D. Dittel B. N. (2005). Gamma delta T cells regulate the extent and duration of inflammation in the central nervous system by a Fas ligand-dependent mechanism. J. Immunol. 174, 4678–4687. 10.4049/jimmunol.174.8.4678 15814692

[B148] Prinz M. Priller J. (2017). The role of peripheral immune cells in the CNS in steady state and disease. Nat. Neurosci. 20, 136–144. 10.1038/nn.4475 28092660

[B149] ProcterT. V.WilliamsA.MontagneA. (2021). Interplay between brain pericytes and endothelial cells in dementia. Am. J. Pathol. 191, 1917–1931. 10.1016/j.ajpath.2021.07.00334329605

[B150] Raffaele S. Boccazzi M. Fumagalli M. (2021). Oligodendrocyte dysfunction in amyotrophic lateral sclerosis: mechanisms and therapeutic perspectives. Cells. 10, 565. 10.3390/cells10030565 33807572 PMC8000560

[B151] Rahimian R. Wakid M. O'Leary L. A. Mechawar N. (2021). The emerging tale of microglia in psychiatric disorders. Neurosci. Biobehav. Rev. 131, 1–29. 10.1016/j.neubiorev.2021.09.023 34536460

[B152] Raj T. Rothamel K. Mostafavi S. Ye C. Lee M. N. Replogle J. M.. (2014). Polarization of the effects of autoimmune and neurodegenerative risk alleles in leukocytes. Science. 344, 519–523. 10.1126/science.1249547 24786080 PMC4910825

[B153] Rannikko E. H. Weber S. S. Kahle P. J. (2015). Exogenous α-synuclein induces toll-like receptor 4 dependent inflammatory responses in astrocytes. BMC Neurosci. 16, 57. 10.1186/s12868-015-0192-0 26346361 PMC4562100

[B154] Ransohoff R. M. (2016). A polarizing question: do M1 and M2 microglia exist? Nat. Neurosci. 19, 987–991. 10.1038/nn.4338 27459405

[B155] Reed-Geaghan E. G. Croxford A. L. Becher B. Landreth G. E. (2020). Plaque-associated myeloid cells derive from resident microglia in an Alzheimer's disease model. J. Exper. Med. 217, e20191374. 10.1084/jem.20191374 31967645 PMC7144522

[B156] Reid-Adam J. Yang N. Song Y. Cravedi P. Li X. M. Heeger P. (2013). Immunosuppressive effects of the traditional Chinese herb Qu Mai on human alloreactive T cells. Am. J. Transplant. 13, 1159–1167. 10.1111/ajt.12180 23433080 PMC3640757

[B157] Renton A. E. Majounie E. Waite A. Simon-Sanchez J. Rollinson S. Gibbs J. R.. (2011). A hexanucleotide repeat expansion in C9ORF72 is the cause of chromosome 9p21-linked ALS-FTD. Neuron. 72, 257–268. 10.1016/j.neuron.2011.09.010 21944779 PMC3200438

[B158] Rentsendorj A. Sheyn J. Fuchs D.-T. Daley D. Salumbides B. C. Schubloom H. E.. (2018). A novel role for osteopontin in macrophage-mediated amyloid-β clearance in Alzheimer's models. Brain Behav. Immun. 67, 163–180. 10.1016/j.bbi.2017.08.019 28860067 PMC5865478

[B159] Reynolds A. D. Banerjee R. Liu J. Gendelman H. E. Mosley R. L. (2007). Neuroprotective activities of CD4+CD25+ regulatory T cells in an animal model of Parkinson's disease. J. Leukoc. Biol. 82, 1083–1094. 10.1189/jlb.0507296 17675560

[B160] Ridler C. (2017). Neuroimmunology: microglia-induced reactive astrocytes - toxic players in neurological disease? Nat. Rev. Neurol. 13, 127. 10.1038/nrneurol.2017.17 28155893

[B161] Ries M. Sastre M. (2016). Mechanisms of Aβ clearance and degradation by glial cells. Front. Aging Neurosci. 8, 160. 10.3389/fnagi.2016.00160 27458370 PMC4932097

[B162] Saab A. S. Tzvetavona I. D. Trevisiol A. Baltan S. Dibaj P. Kusch K.. (2016). Oligodendroglial NMDA receptors regulate glucose import and axonal energy metabolism. Neuron. 91, 119–132. 10.1016/j.neuron.2016.05.016 27292539 PMC9084537

[B163] Sabahi M. Joshaghanian A. Dolatshahi M. Jabbari P. Rahmani F. Rezaei N. (2021). Modification of glial cell activation through dendritic cell vaccination: promises for treatment of neurodegenerative diseases. J. Mol. Neurosci. 71, 1410–1424. 10.1007/s12031-021-01818-6 33713321

[B164] Sabatino J. J. Pröbstel A.-K. Zamvil S. S. (2019). B cells in autoimmune and neurodegenerative central nervous system diseases. Nat. Rev. Neurosci. 20, 728–745. 10.1038/s41583-019-0233-2 31712781

[B165] Sagare A. P. Bell R. D. Zhao Z. Ma Q. Winkler E. A. Ramanathan A.. (2013). Pericyte loss influences Alzheimer-like neurodegeneration in mice. Nat. Commun. 4, 2932. 10.1038/ncomms3932 24336108 PMC3945879

[B166] Saikali P. Antel J. P. Newcombe J. Chen Z. Freedman M. Blain M.. (2007). NKG2D-mediated cytotoxicity toward oligodendrocytes suggests a mechanism for tissue injury in multiple sclerosis. J. Neurosci. 27, 1220–1228. 10.1523/JNEUROSCI.4402-06.2007 17267578 PMC6673175

[B167] Salani F. Sterbini V. Sacchinelli E. Garramone M. Bossù P. (2019). Is innate memory a double-edge sword in Alzheimer's disease? A reappraisal of new concepts and old data. Front. Immunol. 10, 1768. 10.3389/fimmu.2019.01768 31440234 PMC6692769

[B168] Santos E. d.S de Aragão-França L. S. Meira C. S. Cerqueira J. V. Vasconcelos J. F.. (2020). Tolerogenic dendritic cells reduce cardiac inflammation and fibrosis in chronic chagas disease. Front. Immunol. 11, 488. 10.3389/fimmu.2020.00488 32318058 PMC7154094

[B169] Schain A. J. Melo-Carrillo A. Borsook D. Grutzendler J. Strassman A. M. Burstein R. (2018). Activation of pial and dural macrophages and dendritic cells by cortical spreading depression. Ann. Neurol. 83, 508–521. 10.1002/ana.25169 29394508 PMC5965700

[B170] Schirmer L. Rothhammer V. Hemmer B. Korn T. (2013). Enriched CD161high CCR6+ gammadelta T cells in the cerebrospinal fluid of patients with multiple sclerosis. JAMA Neurol. 70, 345–351. 10.1001/2013.jamaneurol.409 23599932

[B171] Schlachetzki J. C. M. Prots I. Tao J. Chun H. B. Saijo K. Gosselin D.. (2018). A monocyte gene expression signature in the early clinical course of Parkinson's disease. Sci. Rep. 8, 10757. 10.1038/s41598-018-28986-7 30018301 PMC6050266

[B172] Schutt C. R. Gendelman H. E. Mosley R. L. (2018). Tolerogenic bone marrow-derived dendritic cells induce neuroprotective regulatory T cells in a model of Parkinson's disease. Molec. Neurodegen. 13, 26. 10.1186/s13024-018-0255-7 29783988 PMC5963189

[B173] Schwartz M. (2017). Can immunotherapy treat neurodegeneration? Science. 357, 254–255. 10.1126/science.aai8231 28729500

[B174] Schwartz M. Deczkowska A. (2016). Neurological disease as a failure of brain-immune crosstalk: the multiple faces of neuroinflammation. Trends Immunol. 37, 668–679. 10.1016/j.it.2016.08.001 27616557

[B175] Shahnawaz M. Mukherjee A. Pritzkow S. Mendez N. Rabadia P. Liu X.. (2020). Discriminating α-synuclein strains in Parkinson's disease and multiple system atrophy. Nature. 578, 273–277. 10.1038/s41586-020-1984-7 32025029 PMC7066875

[B176] Shalash A. Salama M. Makar M. Roushdy T. Elrassas H. H. Mohamed W.. (2017). Elevated serum α-synuclein autoantibodies in patients with Parkinson's disease relative to Alzheimer's disease and controls. Front. Neurol. 8, 720. 10.3389/fneur.2017.00720 29312137 PMC5744443

[B177] Shechter R. Miller O. Yovel G. Rosenzweig N. London A. Ruckh J.. (2013). Recruitment of beneficial M2 macrophages to injured spinal cord is orchestrated by remote brain choroid plexus. Immunity. 38, 555–569. 10.1016/j.immuni.2013.02.012 23477737 PMC4115271

[B178] Sheean R. K. McKay F. C. Cretney E. Bye C. R. Perera N. D. Tomas D.. (2018). Association of regulatory t-cell expansion with progression of amyotrophic lateral sclerosis: a study of humans and a transgenic mouse model. JAMA Neurol. 75, 681–689. 10.1001/jamaneurol.2018.0035 29507931 PMC5885208

[B179] Snaidero N. Schifferer M. Mezydlo A. Zalc B. Kerschensteiner M. Misgeld T. (2020). Myelin replacement triggered by single-cell demyelination in mouse cortex. Nat. Commun. 11, 4901. 10.1038/s41467-020-18632-0 32994410 PMC7525521

[B180] Sommer A. Marxreiter F. Krach F. Fadler T. Grosch J. Maroni M.. (2018). Th17 lymphocytes induce neuronal cell death in a human iPSC-based model of Parkinson's disease. Cell Stem. Cell. 23, 93–107. 10.1016/j.stem.2018.06.015 29979986

[B181] Song S. Miranda C. J. Braun L. Meyer K. Frakes A. E. Ferraiuolo L.. (2016). Major histocompatibility complex class I molecules protect motor neurons from astrocyte-induced toxicity in amyotrophic lateral sclerosis. Nat. Med. 22, 397–403. 10.1038/nm.4052 26928464 PMC4823173

[B182] Sonninen T.-M. Hämäläinen R. H. Koskuvi M. Oksanen M. Shakirzyanova A. Wojciechowski S.. (2020). Metabolic alterations in Parkinson's disease astrocytes. Sci. Rep. 10, 14474. 10.1038/s41598-020-71329-8 32879386 PMC7468111

[B183] Späni C. Suter T. Derungs R. Ferretti M. T. Welt T. Wirth F.. (2015). Reduced β-amyloid pathology in an APP transgenic mouse model of Alzheimer's disease lacking functional B and T cells. Acta Neuropathol. Commun. 3, 71. 10.1186/s40478-015-0251-x 26558367 PMC4642668

[B184] Spiller K. J. Cheung C. J. Restrepo C. R. Kwong L. K. Stieber A. M. Trojanowski J. Q.. (2016). Selective motor neuron resistance and recovery in a new inducible mouse model of TDP-43 proteinopathy. J .Neurosci. 36, 7707–7717. 10.1523/JNEUROSCI.1457-16.2016 27445147 PMC6705561

[B185] Spiller K. J. Restrepo C. R. Khan T. Dominique M. A. Fang T. C. Canter R. G.. (2018). Microglia-mediated recovery from ALS-relevant motor neuron degeneration in a mouse model of TDP-43 proteinopathy. Nat. Neurosci. 21, 329–340. 10.1038/s41593-018-0083-7 29463850 PMC5857237

[B186] Sreedharan J. Blair I. P. Tripathi V. B. Hu X. Vance C. Rogelj B.. (2008). TDP-43 mutations in familial and sporadic amyotrophic lateral sclerosis. Science. 319, 1668–1672. 10.1126/science.1154584 18309045 PMC7116650

[B187] Strecker J.-K. Schmidt A. Schäbitz W.-R. Minnerup J. (2017). Neutrophil granulocytes in cerebral ischemia - Evolution from killers to key players. Neurochem. Int. 107, 117–126. 10.1016/j.neuint.2016.11.006 27884770

[B188] Subramaniam S. R. Federoff H. J. (2017). Targeting microglial activation states as a therapeutic avenue in Parkinson's disease. Front. Aging Neurosci. 9, 176. 10.3389/fnagi.2017.00176 28642697 PMC5463358

[B189] Taherzadeh-Fard E. Saft C. Akkad D. A. Wieczorek S. Haghikia A. Chan A.. (2011). PGC-1alpha downstream transcription factors NRF-1 and TFAM are genetic modifiers of Huntington disease. Molec. Neurodegen. 6, 32. 10.1186/1750-1326-6-32 21595933 PMC3117738

[B190] Tansey M. G. Romero-Ramos M. (2019). Immune system responses in Parkinson's disease: early and dynamic. Eur. J. Neurosci. 49, 364–383. 10.1111/ejn.14290 30474172 PMC6391192

[B191] Thadathil N. Nicklas E. H. Mohammed S. Lewis T. L. Richardson A. Deepa S. S. (2021). Necroptosis increases with age in the brain and contributes to age-related neuroinflammation. Geroscience. 43, 2345–2361. 10.1007/s11357-021-00448-5 34515928 PMC8599532

[B192] Thonhoff J. R. Simpson E. P. Appel S. H. (2018). Neuroinflammatory mechanisms in amyotrophic lateral sclerosis pathogenesis. Curr. Opin. Neurol. 31, 635–639. 10.1097/WCO.0000000000000599 30048339

[B193] Trias E. Ibarburu S. Barreto-Núñez R. Varela V. Moura I. C. Dubreuil P.. (2017). Evidence for mast cells contributing to neuromuscular pathology in an inherited model of ALS. JCI Insight. 2, e95934. 10.1172/jci.insight.95934 29046475 PMC5846907

[B194] Trias E. King P. H. Si Y. Kwon Y. Varela V. Ibarburu S.. (2018). Mast cells and neutrophils mediate peripheral motor pathway degeneration in ALS. JCI Insight. 3, e123249. 10.1172/jci.insight.123249 30282815 PMC6237484

[B195] Tunca C. Akçimen F. Coşkun C. Gündogdu-Eken A. Kocoglu C. Çevik B.. (2018). ERLIN1 mutations cause teenage-onset slowly progressive ALS in a large Turkish pedigree. Eur. J. Hum. Genet. 26, 745–748. 10.1038/s41431-018-0107-5 29453415 PMC5945623

[B196] Uemura M. T. Maki T. Ihara M. Lee V. M. Y. Trojanowski J. Q. (2020). Brain Microvascular Pericytes in Vascular Cognitive Impairment and Dementia. Front. Aging Neurosci. 12, 80. 10.3389/fnagi.2020.00080 32317958 PMC7171590

[B197] Urban S. L. Jensen I. J. Shan Q. Pewe L. L. Xue H.-H. Badovinac V. P.. (2020). Peripherally induced brain tissue-resident memory CD8 T cells mediate protection against CNS infection. Nat. Immunol. 21, 938–949. 10.1038/s41590-020-0711-8 32572242 PMC7381383

[B198] Vainchtein I. D. Molofsky A. V. (2020). Astrocytes and Microglia: In Sickness and in Health. Trends Neurosci. 43, 144–154. 10.1016/j.tins.2020.01.003 32044129 PMC7472912

[B199] Vance C. Rogelj B. Hortobágyi T. De Vos K. J. Nishimura A. L. Sreedharan J.. (2009). Mutations in FUS, an RNA processing protein, cause familial amyotrophic lateral sclerosis type 6. Science. 323, 1208–1211. 10.1126/science.1165942 19251628 PMC4516382

[B200] Varatharaj A. Galea I. (2017). The blood-brain barrier in systemic inflammation. Brain Behav. Immun. 60, 1–12. 10.1016/j.bbi.2016.03.010 26995317

[B201] Vedam-Mai V. (2021). Harnessing the immune system for the treatment of Parkinson's disease. Brain Res. 1758, 147308. 10.1016/j.brainres.2021.147308 33524380

[B202] Voskoboinik I. Whisstock J. C. Trapani J. A. (2015). Perforin and granzymes: function, dysfunction and human pathology. Nat. Rev. Immunol. 15, 388–400. 10.1038/nri3839 25998963

[B203] Wang X. Sun G. Feng T. Zhang J. Huang X. Wang T.. (2019). Sodium oligomannate therapeutically remodels gut microbiota and suppresses gut bacterial amino acids-shaped neuroinflammation to inhibit Alzheimer's disease progression. Cell Res. 29, 787–803. 10.1038/s41422-019-0216-x 31488882 PMC6796854

[B204] Wang Y. Wang Z. Wang Y. Li F. Jia J. Song X.. (2018). The Gut-Microglia Connection: Implications for Central Nervous System Diseases. Front. Immunol. 9, 2325. 10.3389/fimmu.2018.02325 30344525 PMC6182051

[B205] Weiss A. Träger U. Wild E. J. Grueninger S. Farmer R. Landles C.. (2012). Mutant huntingtin fragmentation in immune cells tracks Huntington's disease progression. J. Clin. Invest. 122, 3731–3736. 10.1172/JCI64565 22996692 PMC3461928

[B206] Wo J. Zhang F. Li Z. Sun C. Zhang W. Sun G. (2020). The Role of Gamma-Delta T Cells in Diseases of the Central Nervous System. Front. Immunol. 11, 580304. 10.3389/fimmu.2020.580304 33193380 PMC7644879

[B207] Xu E. Boddu R. Abdelmotilib H. A. Sokratian A. Kelly K. Liu Z.. (2022). Pathological α-synuclein recruits LRRK2 expressing pro-inflammatory monocytes to the brain. Molec. Neurodegen. 17, 7. 10.1186/s13024-021-00509-5 35012605 PMC8751347

[B208] Xu W. L. Lau Z. W. X. Fulop T. Larbi A. (2020). The aging of gamma delta T cells. Cells. 9, 1181. 10.3390/cells9051181 32397491 PMC7290956

[B209] Yang J. Kumar A. Vilgelm A. E. Chen S.-C. Ayers G. D. Novitskiy S. V.. (2018). Loss of CXCR4 in myeloid cells enhances antitumor immunity and reduces melanoma growth through NK Cell and FASL mechanisms. Cancer Immunol. Res. 6, 1186–1198. 10.1158/2326-6066.CIR-18-0045 30108045 PMC6170679

[B210] Yu J. Guo M. Li Y. Zhang H. Chai Z. Wang Q.. (2019). Astragaloside IV protects neurons from microglia-mediated cell damage through promoting microglia polarization. Folia. Neuropathol. 57, 170–181. 10.5114/fn.2019.86299 31556576

[B211] Yun S. P. Kam T.-I. Panicker N. Kim S. Oh Y. Park J.-S.. (2018). Block of A1 astrocyte conversion by microglia is neuroprotective in models of Parkinson's disease. Nat. Med. 24, 931–938. 10.1038/s41591-018-0051-5 29892066 PMC6039259

[B212] Zamudio F. Loon A. R. Smeltzer S. Benyamine K. Navalpur Shanmugam N. K. Stewart N. J. F.. (2020). TDP-43 mediated blood-brain barrier permeability and leukocyte infiltration promote neurodegeneration in a low-grade systemic inflammation mouse model. J. Neuroinflammation. 17, 283. 10.1186/s12974-020-01952-9 32979923 PMC7519496

[B213] Zeis T. Enz L. Schaeren-Wiemers N. (2016). The immunomodulatory oligodendrocyte. Brain Res. 1641, 139–148. 10.1016/j.brainres.2015.09.021 26423932

[B214] Zella M. A. S. Metzdorf J. Ostendorf F. Maass F. Muhlack S. Gold R.. (2019). Novel Immunotherapeutic Approaches to Target Alpha-Synuclein and Related Neuroinflammation in Parkinson's Disease. Cells. 8, 105. 10.3390/cells8020105 30708997 PMC6406239

[B215] Zella S. M. A. Metzdorf J. Ciftci E. Ostendorf F. Muhlack S. Gold R.. (2019). Emerging immunotherapies for Parkinson disease. Neurol. Ther. 8, 29–44. 10.1007/s40120-018-0122-z 30539376 PMC6534677

[B216] Zenaro E. Pietronigro E. Della Bianca V. Piacentino G. Marongiu L. Budui S.. (2015). Neutrophils promote Alzheimer's disease-like pathology and cognitive decline via LFA-1 integrin. Nat. Med. 21, 880–886. 10.1038/nm.3913 26214837

[B217] Zhang Y. Fung I. T. H. Sankar P. Chen X. Robison L. S. Ye L.. (2020). Depletion of NK cells improves cognitive function in the Alzheimer disease mouse model. J. Immunol. 205, 502–510. 10.4049/jimmunol.2000037 32503894 PMC7343613

[B218] Zhao W. Beers D. R. Bell S. Wang J. Wen S. Baloh R. H.. (2015). TDP-43 activates microglia through NF-κB and NLRP3 inflammasome. Exp. Neurol. 273, 24–35. 10.1016/j.expneurol.2015.07.019 26222336

[B219] Zhou T. Zheng Y. Sun L. Badea S. R. Jin Y. Liu Y.. (2019). Microvascular endothelial cells engulf myelin debris and promote macrophage recruitment and fibrosis after neural injury. Nat. Neurosci. 22, 421–435. 10.1038/s41593-018-0324-9 30664769 PMC6913093

[B220] Zhou Z. He H. Wang K. Shi X. Wang Y. Su Y.. (2020). Granzyme A from cytotoxic lymphocytes cleaves GSDMB to trigger pyroptosis in target cells. Science. 368, eaaz7548. 10.1126/science.aaz7548 32299851

[B221] Zondler L. Müller K. Khalaji S. Bliederhäuser C. Ruf W. P. Grozdanov V.. (2016). Peripheral monocytes are functionally altered and invade the CNS in ALS patients. Acta Neuropathol. 132, 391–411. 10.1007/s00401-016-1548-y 26910103

